# Targeting Ferroptosis Pathway to Combat Therapy Resistance and Metastasis of Cancer

**DOI:** 10.3389/fphar.2022.909821

**Published:** 2022-06-30

**Authors:** Xuan Liu, Yiqian Zhang, Xuyi Wu, Fuyan Xu, Hongbo Ma, Mengling Wu, Yong Xia

**Affiliations:** ^1^ Department of Rehabilitation Medicine, West China Hospital, Sichuan University, Chengdu, China; ^2^ West China School of Pharmacy, Sichuan University, Chengdu, China; ^3^ Key Laboratory of Rehabilitation Medicine in Sichuan Province/Rehabilitation Medicine Research Institute, Chengdu, China

**Keywords:** ferroptosis, drug resistance, cancer, metastasis, peroxidation

## Abstract

Ferroptosis is an iron-dependent regulated form of cell death caused by excessive lipid peroxidation. This form of cell death differed from known forms of cell death in morphological and biochemical features such as apoptosis, necrosis, and autophagy. Cancer cells require higher levels of iron to survive, which makes them highly susceptible to ferroptosis. Therefore, it was found to be closely related to the progression, treatment response, and metastasis of various cancer types. Numerous studies have found that the ferroptosis pathway is closely related to drug resistance and metastasis of cancer. Some cancer cells reduce their susceptibility to ferroptosis by downregulating the ferroptosis pathway, resulting in resistance to anticancer therapy. Induction of ferroptosis restores the sensitivity of drug-resistant cancer cells to standard treatments. Cancer cells that are resistant to conventional therapies or have a high propensity to metastasize might be particularly susceptible to ferroptosis. Some biological processes and cellular components, such as epithelial–mesenchymal transition (EMT) and noncoding RNAs, can influence cancer metastasis by regulating ferroptosis. Therefore, targeting ferroptosis may help suppress cancer metastasis. Those progresses revealed the importance of ferroptosis in cancer, In order to provide the detailed molecular mechanisms of ferroptosis in regulating therapy resistance and metastasis and strategies to overcome these barriers are not fully understood, we described the key molecular mechanisms of ferroptosis and its interaction with signaling pathways related to therapy resistance and metastasis. Furthermore, we summarized strategies for reversing resistance to targeted therapy, chemotherapy, radiotherapy, and immunotherapy and inhibiting cancer metastasis by modulating ferroptosis. Understanding the comprehensive regulatory mechanisms and signaling pathways of ferroptosis in cancer can provide new insights to enhance the efficacy of anticancer drugs, overcome drug resistance, and inhibit cancer metastasis.

## 1 Introduction

Cancer is the second leading cause of death globally and is characterized by the uncontrolled growth of abnormal cells, invading adjacent sites and spreading to other organs. The latter process, called metastasis, is the main cause of cancer-related death. Induction of apoptosis with anticancer drugs (including targeted therapy, chemotherapy, and immunotherapy) or radiation therapy is the main strategy for the treatment of cancer, but innate and acquired resistance can reduce the therapeutic effect (Yang G. et al., 2021). Induction of non-apoptotic types of cell death could open new avenues to eliminate cancer cells and limit drug resistance. Ferroptosis is morphologically characterized by decreased mitochondrial volume, reduction or disappearance of mitochondrial cristae, and rupture of the plasma membrane and mitochondrial membrane (Dixon et al., 2012). As a newly discovered programmed cell death pathway, ferroptosis is defined as an iron-catalyzed regulated necrosis that occurs through excessive peroxidation of polyunsaturated fatty acids (PUFAs) (Dixon et al., 2012).

Recent studies have shown that ferroptosis is involved in the antitumor effects of many anticancer drugs/radiotherapy and resistance to various treatments, but the specific molecular mechanism is still not fully understood (Conrad et al., 2016). For example, approved drugs (such as sulfasalazine and artemisinin) experimental reagents (such as erastin and RSL3) and ionizing radiation can induce ferroptosis (Chen et al., 2021a). But the specific molecular mechanism is still not fully understood (Conrad et al., 2016). Cancer metastasis and resistance to therapy are two major obstacles to improving patient survival and quality of life. A better understanding of the molecular mechanisms of ferroptosis in these two processes and exploring how to target this death process could provide useful guidance for improving patient outcomes. Therefore, we described the mechanisms and regulators of ferroptosis and elucidated the mechanism by which ferroptosis is involved in drug resistance and cancer metastasis and summarized strategies to target ferroptosis to combat drug resistance and cancer metastasis. Finally, we provide an outlook for future research on ferroptosis in cancer.

## 2 Molecular Mechanism of Ferroptosis

Ferroptosis was initially proposed in the precision medicine of Ras-mutant tumors (Dolma et al., 2003; Yang and Stockwell, 2008). Serving as a proto-oncogene, Ras mutations are frequently detected in human cancers that cause drug resistance. Small molecule compounds erastin and RAS-selective lethal 3 (RSL3) can selectively kill Ras mutant cancer cells rather than cancer cells carrying wild-type RAS (Zhao et al., 2022). Later, the anticancer activities of erastin and RSL3 were validated to be dependent on a novel iron-dependent programmed cell death, known as ferroptosis (Dixon et al., 2012). Ferroptosis used to be considered a type of programmed cell death that differs from conventional cell death, such as apoptosis, necrosis, and autophagy, and the latest evidence has revealed their close interaction. Direct triggers for ferroptosis remain unclear, and it is believed that ferroptosis is a highly complicated and strictly regulated process involving iron accumulation, lipid peroxidation, and mitochondrial membrane rupture. The process of ferroptosis can be influenced by epigenetic, transcriptional, and posttranscriptional and posttranslational regulation (Dai et al., 2020; Wu et al., 2020). The occurrence of ferroptosis is iron-dependent (Hassannia et al., 2019), which is initiated after the impaired capacities to eliminate free radicals in the human body (Kuang et al., 2020b). Therefore, reactive oxygen species (ROS) and lipid peroxidation are of great significance in the regulation of ferroptosis (Dixon et al., 2012; Hayano et al., 2016).

### 2.1 ROS Production

ROS act as signaling molecules to trigger various types of cell death, including ferroptosis (Dixon et al., 2012). ROS and lipid peroxidation are critical for ferroptosis (Kuang et al., 2020b), which requires the accumulation of ROS throughout the whole process (Dixon et al., 2012).

#### 2.1.1 Iron-Induced Production of ROS

Hydroxyl radicals are the most chemically active ROS involved in ferroptosis and are highly mobile and water soluble. Fenton and Fenton-like reactions are the main source of hydroxyl radicals, which are mainly involved in the reaction between H_2_O_2_ and transition metals such as labile iron (Fe^2+^) (Fenton, 1894; Ayala et al., 2014). The intracellular free iron level is dynamically regulated by iron absorption, storage, transport, and extracellular transport. Any intracellular accumulation of iron would affect the iron level and ROS production, which ultimately influences the sensitivity to ferroptosis (Chen et al., 2021c). In animal models, multilevel interventions, such as increasing iron absorption, increasing iron storage, and limiting iron efflux, lead to iron accumulation, and eventually, the integrated signaling pathways contribute to mediating ferroptosis (Chen X. et al., 2020).

#### 2.1.2 NOX-Induced Production of ROS

Phagocytes such as macrophages and dendritic cells (DCs) are able to express nicotinamide adenine dinucleotide phosphate (NADPH) oxidases (NOXs), which contribute to the production of ROS during the process of ferroptosis by generating ${O_{2}}^{\bar{\cdot }}$ . Other cells are also capable of expressing NOXs by generating ${O_{2}}^{\bar{\cdot }}$ or H_2_O_2_ by transporting electrons across the membrane. There are seven NOXs, including five NOX proteins (NOX1, CYBB/NOX2, NOX3, NOX4, and NOX5) and two dual oxidases (DUOX1 and DUOX2). ROS produced by nitrogen oxides are widely participated in various physiological and pathological states, such as development, infection, immunity, and cell death (Bedard and Krause, 2007). NOX is also involved in inducing apoptosis as an important regulator of lipid raft–derived signals (Jin et al., 2011). ROS produced by NOX1, CYBB, and NOX4 are also involved in the ferroptosis in cancer cells, indicating that NOX plays a broad role in programmed cell death (Xie et al., 2017; Yang et al., 2019). Oncogenes and tumor suppressor genes can affect NOX activity in ferroptosis. For example, inactivation of the tumor suppressor gene p53 inhibits nuclear accumulation of dipeptidyl peptidase-4 (DPP4/CD26), thereby stimulating plasma membrane–associated DPP4-dependent lipid peroxidation. Formation of the DPP4-NOX1 complex leads to cell death (Xie et al., 2017). During Ras activation, NOX1-induced ROS promote iron ptosis by activating the ERK signaling pathway (Yagoda et al., 2007; Adachi et al., 2008). Currently, more efforts are needed to explore the potential signaling pathways through NOX involved in ferroptosis in cancer cells.

### 2.2 Lipid Peroxidation

Oxidative stress is caused by imbalanced scavenging and the production of free radicals. ROS-mediated lipid peroxidation is a key step leading to ferroptosis, including enzymatic and non-enzymatic lipid peroxidation. PUFAs, especially arachidonic acids and adrenic acids, are the most prone to lipid peroxidation, which damages the lipid bilayer and affects membrane function. Lysophosphatidylcholine acyltransferase 3 (LPCAT3) and acyl-CoA synthetase long-chain 4 (ACSL4) are necessary for the biosynthesis and remodeling of PUFAs in the cell membrane. The latter catalyzes the binding of free arachidonic acid or epinephrine to CoA to form derivatives AA-CoA or AdA-CoA, which are then esterified by LPCAT3 to form membrane phosphatidylethanolamine to form AA-PE or AdA-PE (Yuan et al., 2016; Doll et al., 2017). ACSL3 protects cancer cells from ferroptosis by converting monounsaturated fatty acids (MUFA) to acyl-CoA esters and binding to membrane phospholipids. AMP-activated protein kinase (AMPK)–mediated beclin 1 phosphorylation promotes ferroptosis by inhibiting glutathione (GSH) production, while AMPK-mediated acetyl-CoA carboxylase (ACAC) phosphorylation inhibits ferroptosis by limiting PUFA production.

#### 2.2.1 ROS-Induced Non-Enzymatic Lipid Peroxidation

Non-enzymatic lipid peroxidation or lipid autooxidation is a chain reaction driven by free radicals, in which ROS trigger the oxidation of PUFAs. The formation of lipid free radicals by the binding of hydroxyl radicals to PUFAs is the first step of iron-involved lipid peroxidation. Later, lipid free radicals abstract hydrogens from the adjacent polyunsaturated fatty acids, which results in the formation of PLOOH and new lipid radicals. As a result, a novel lipid radical chain reaction occurs. With the involvement of ferrous ions, lipid hydroperoxide is converted into alkoxyl radicals (LO•), which react with adjacent PUFAs to initiate another lipid radical chain reaction. Catalyzed by iron and oxygen, this autoamplifying process leads to membrane disruption and cell death when molecules that prevent lipid peroxidation are inactivated (Reis and Spickett, 2012; Doll et al., 2017).

#### 2.2.2 ROS-Induced Enzymatic Lipid Peroxidation

ROS can also be catalyzed by ALOX, a dioxygenase that contains nonheme iron. The ALOX family consists of six members, namely, ALOX3, ALOX5, ALOX12, ALOX12B, ALOX15, and ALOX15B. They oxidize polyunsaturated fatty acids, especially arachidonic acid (AA) and adrenoic acid (AdA), in a tissue- or cell-dependent manner. Linoleic acid (LA) and AA are two common substrates of ALOX in mammalian cells. ALOX5 contributes to the synthesis of 5-hydropereicosentaenoic acid (5-hpete) through the oxidation of AA at carbon 5 (Kuhn et al., 2015). ALOX12 and ALOX15 synthesize 12-HPETE and 15-Hpete from AA, 9-hpode, and LA (Jung et al., 1985). LOX12 and LOX15 can directly oxidize AA-containing phospholipids (PLs), whereas ALOX5 needs the cellulolytic phospholipase A2 (cPLA2) to first hydrolyze the esterified AA on the membrane (Jung et al., 1985; Takahashi et al., 1993).

### 2.3 Oxidative Stress in Ferroptosis

Oxidative stress in ferroptosis is a multilevel process. Antioxidants, such as GSH, coenzyme Q10 (CoQ10), and tetrahydrobiopterin (BH4), are the main fighters against ferroptosis and are closely linked with multiple enzymes or proteins. Some antioxidant proteins, such as peroxiredoxins (PRDXs) and thioredoxin, can also prevent ferroptosis (Dolma et al., 2003; Lovatt et al., 2020). Therefore, the oxidative stress mechanism in ferroptosis remains complicated.

## 3 Regulatory Mechanism of Ferroptosis

**FIGURE 1:**
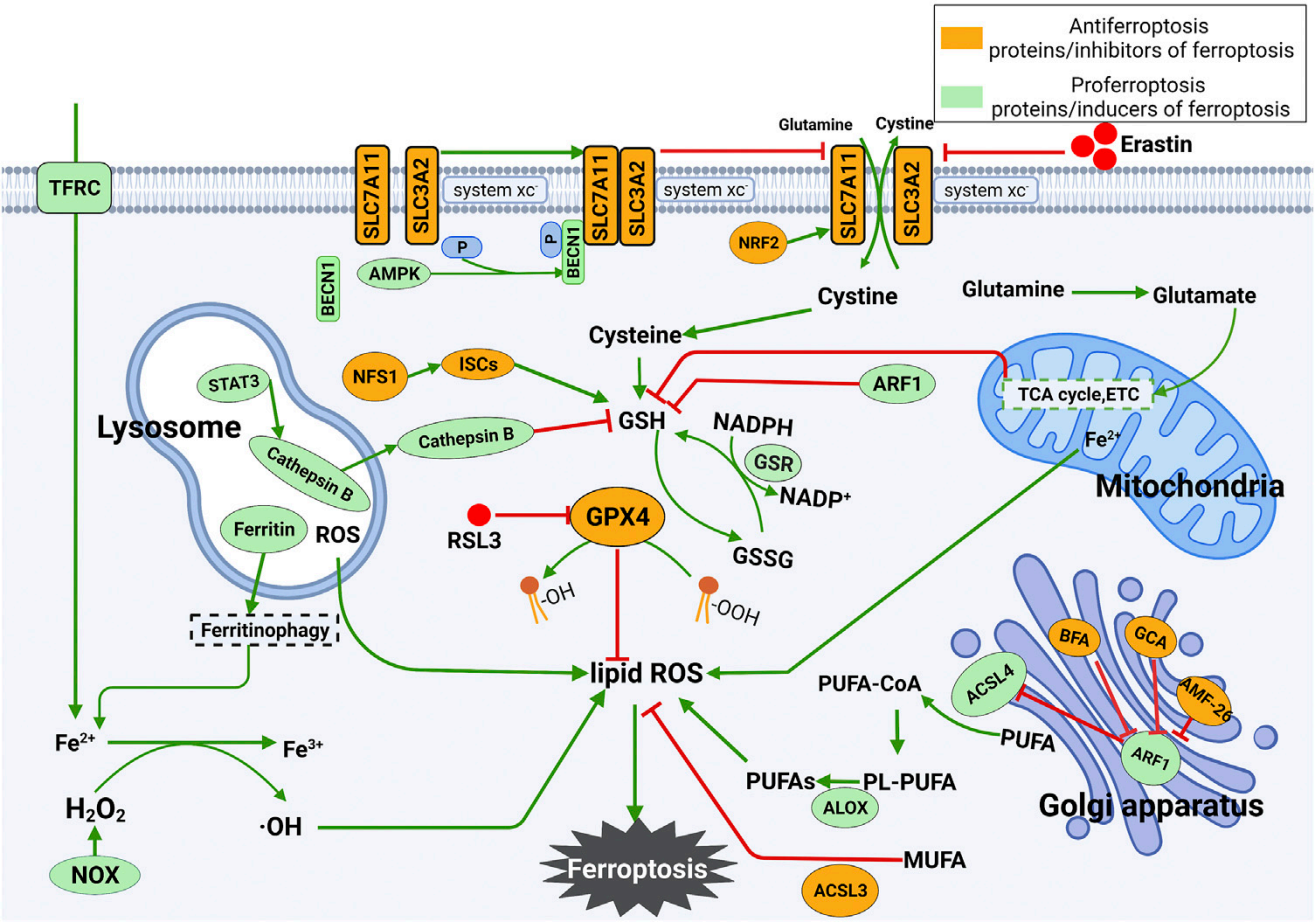
Mechanism underlying the occurrence and regulation of ferroptosis. **(1)** Ferroptosis is mainly caused by lipid peroxidation. ROS leading to ferroptosis are produced by the iron-dependent Fenton reaction, mitochondrial electron transport chain or NOX proteins. Ferroptosis can be triggered by enhancing the synthesis of lipid ROS. **(2)** Inhibition of SLC7A11 deprives cells of cysteine, resulting in the loss of GSH and inactivation of GPX4. The latter further leads to the accumulation of lipid ROS and ferroptosis. The tricarboxylic acid cycle (TCA cycle) and electron carriers (ETC) in mitochondria stimulate GSH deficiency, thus leading to ferroptosis. The release of Fe^2+^ in mitochondria increases the level of free Fe^2+^ in cells and eventually promotes the production of lipid ROS. Lysosomal ROS contribute to the production of lipid ROS. In lysosomes, STAT3-mediated expression of cathepsin B is essential for ferroptosis via the MEK-ERK signaling pathway. In the Golgi apparatus, the Golgi stress response can inhibit ARF1, which is an inhibitor of GSH and ACSL4 and an activator of SLC7A11. Silencing ARF1 promotes ferroptosis by increasing cellular ROS levels.

There are two central biochemical events in iron death leading to ferroptosis, including intracellular iron accumulation and lipid peroxidation. In addition to the occurrence mechanism, the regulatory mechanism of ferroptosis is also complicated. In the following sections, the main regulatory mechanisms and regulators underlying ferroptosis will be summarized. The occurrence and regulatory mechanism of ferroptosis are shown in Figure 1.

### 3.1 Novel Roles of Different Organelles in the Regulation of Ferroptosis

Organelles are tiny units necessary for normal cell function. Notably, organelle dysfunction responsive to stress would stimulate cell death (Xu et al., 2019). Ferroptosis is a strictly regulated process involving various signaling pathways and regulators in different organelles, including mitochondria, lysosomes, and the Golgi apparatus (Chen et al., 2021b).

In the following sections, we will review the specific functions of several important organelles in the regulation of ferroptosis.

#### 3.1.1 Role of Mitochondria in Ferroptosis

Mitochondria generate most of the chemical energy required for biochemical reactions of cells and store it. Cells experiencing ferroptosis typically exhibit a reduction in mitochondrial cristae, decrease in mitochondrial size, increased mitochondrial membrane density, and increased permeability, suggesting the occurrence of mitochondrial dysfunction during ferroptosis (Dixon et al., 2012).

Mitochondria are an important source of ROS during oxidative phosphorylation in most mammalian cells. Local production of ROS not only leads to damage of mitochondria but also affects the redox status of the remaining components of the cell (Friedmann Angeli et al., 2014). Since mitochondrial ROS mainly induce apoptosis, they used to be considered irrelevant to ferroptosis. Later, they were validated to target ROS scavengers such as Mito-TEMPO (MT) and Mitoquinone (MitoQ), which are able to inhibit ferroptosis in multiple types of cells (e.g., cancer cells, cardiomyocytes, and hippocampal neurons) (Jelinek et al., 2018; Fang et al., 2019). Inhibition of the mitochondrial electron transport chain or tricarboxylic acid (TCA) cycle inhibits ferroptosis induced by cysteine deprivation. Mitochondrial fatty acid metabolism genes, including acyl-CoA synthase family member 2 (ACSF2) and citrate synthase (CS), may be required for erastin-induced ferroptosis (Xu et al., 2019). Impaired mitochondrial iron metabolism also promotes ferroptosis. Free extracellular iron is taken up by cells and transported into mitochondria, where it is partially used for the synthesis of heme and iron-sulfur clusters (ISC) and the remainder is stored in mitochondrial ferritin. High levels of iron in the mitochondria can mediate the production of ROS or interfere with the normal function of enzymes (Chen et al., 2021b). Therefore, mitochondria are closely associated with the induction of ferroptosis.

#### 3.1.2 Role of Lysosomes in Ferroptosis

Lysosomes also participate in the induction of ferroptosis. They are acidic membrane-bound organelles that promote ferroptosis by activating autophagy and releasing lysosome cathepsin B (CTSB). In HT1080 cells, lysosomes are the major source of ROS for erastin-induced ferroptosis. In addition, lysosomes can also affect intracellular iron supply by attenuating intracellular transferrin transport or autophagic degradation of ferritin.

Studies on the mechanism of ferroptosis have identified the release of lysosomal proteases as a cause of ferroptosis. Inhibition of lysosomal proteases, particularly CTSB, reduces cellular susceptibility to erastin-induced ferroptosis. STAT3 regulates the expression level of CTSB in human pancreatic ductal adenocarcinoma cell lines, and it promotes ferroptosis through this pathway. Nuclear translocation of lysosomal CTSB has been reported to lead to DNA damage and subsequent interferon-response stimulator of interferon response cGAMP interactor 1 (STING1)-dependent ferroptosis (Kuang et al., 2020a). In addition to inhibiting lysosomal function, silencing of cathepsin limits erastin-induced ferroptosis in cells (Kuang et al., 2020a; Nagakannan et al., 2021). Ferroptosis is part of cellular autophagy and is executed by sequential contribution of autophagy-related (ATG) proteins in a hierarchical manner (Klionsky et al., 2021), while lysosomes are the main organelles of autophagic degradation of protein aggregates and have an important role in ferroptosis (Radisky and Kaplan, 1998; De Domenico et al., 2006). Knockdown of genes such as ATG3, ATG5, ATG7, ATG13, and ATG6 (also known as BECN1) inhibited iron uptake and thus ferroptosis in many types of cancer cells (Gao et al., 2016; Hou et al., 2016). In contrast, knockdown of ATG2A promoted ferroptosis in the cervical cancer cell line HeLa by increasing uptake of Fe^2+^ (Xiong et al., 2021).

The latest study has developed drugs localized to lysosomes that inhibit or promote ferroptosis. For example, N, N-dimethylaniline derivatives localize to late endosomes and lysosomes, which are able to prevent ferroptosis (Hirata et al., 2021). Dichloroacetate promotes ferroptosis in colorectal cancer cells by chelating iron in lysosomes (Sun J. et al., 2021). Taken together, lysosomes are promising targets for ferroptosis.

#### 3.1.3 Golgi Stress Participates in the Occurrence of Ferroptosis

Golgi stress plays an important role in ferroptosis (Alborzinia et al., 2018). Some Golgi stressors, such as AMF-26 (also known as M-COPA), golgicide A (GCA), and brefeldin A (BFA), can trigger ferroptosis. Ferroptosis inhibitors protect cells by preventing the rupture of Golgi apparatus and inhibit protein secretion to fight against Golgi stressors (Wu et al., 2020). Erastin at a sublethal concentration is sufficient to alleviate lipid peroxidation caused by Golgi stress. The trans-sulfuration pathway is responsible for limiting ferroptosis, serving as a compensation for cysteine supply after oxidative stress (Garg et al., 2011; Hayano et al., 2016). The coinduction of pharmacological inhibitors of the trans-sulfuration pathway and low-dose erastin abolishes the effect of erastin binding to Golgi stressors on promoting cell survival. Therefore, it is believed that the Golgi apparatus is involved in the redox reaction and regulation of ferroptosis.

### 3.2 Regulators of Ferroptosis

#### 3.2.1 Role of GPX4 in Ferroptosis

Glutathione peroxidases (GPXs) can relieve ferroptosis caused by peroxidative damage to the cell membrane (Bochkov et al., 2010). Among them, GPX4 is an important member in GSH metabolism, which maintains the homeostasis of intracellular lipid peroxides. GPX4 can reduce phospholipid hydroperoxide (AA/AdA-PE-OOH) to the corresponding phospholipid alcohol (PLOH) by exerting its enzymatic activity, thereby interrupting the radical-chain reaction and inhibiting the accumulation of intracellular lipid peroxides (Tang D. et al., 2021). The detoxifying ability of GPX4 exists even when hydroperoxides are inserted into biomembranes or lipoproteins. Therefore, GPX4 is considered the only GPX capable of protecting biofilms from peroxidation. In addition, GPX4 can also maintain the stability of the bilayer lipid membrane (Maiorino et al., 2018). The depletion of intracellular GSH inactivates GPXs and induces ferroptosis (Yang et al., 2014), suggesting that GSH is a cofactor for GPX4 to catalyze the production of phospholipid alcohol from peroxides (Xu et al., 2019).

Although GPX4 inhibition is an important downstream signal, it is not necessary for initiating ferroptosis. For example, TP53 induces ferroptosis by downregulating SLC7A11, in which the inhibition of GPX4 is not necessary (Chu et al., 2019). The deficiency of GPX4 or SLC7A11 significantly increases cell resistance to Golgi stress-induced ferroptosis, suggesting complicated regulatory mechanisms in ferroptosis (Alborzinia et al., 2018; Tang D. et al., 2021).

#### 3.2.2 SLC7A11 is a Functional Light Chain Subunit of the Cystine/Glutamate Antiporter That Takes up Extracellular Cystine

Low GSH levels, insufficient supply of cysteine, or GPX4 inhibition caused by phospholipid hydroperoxides (PLOOHs) are one of the important mechanisms to initiate ferroptosis (Dixon et al., 2012; Homma et al., 2019). Previous evidence has shown that the cystine/glutamate antiporter SLC7A11 is associated with the initiation of ferroptosis. SLC7A11 mediates the uptake of extracellular cystine in exchange for GSH, which prevents the accumulation of lipid peroxides and ferroptosis. It is also a key regulator of iron overload/ferroptosis (Liu L. et al., 2021). Iron overload refers to the excessive deposition of iron in the body which leads to structural damage and dysfunction of vital organs. Imbalance in iron homeostasis may be involved in the development of certain cancers and can also lead to tumor cell death. In myelodysplastic syndrome (MDS) and acute myeloid leukemia (AML), iron overload contributes to the production of ROS, which are involved in leukemic transformation by producing mutagenic and genotoxic substances (Cadet and Wagner, 2013). On the other hand, iron overload can lead to ferroptosis through ROS and consequent lipid peroxidation in extreme cases (Dixon and Stockwell, 2014). Iron mediates the expression level of SLC7A11 through the ROS-Nrf2-ARE axis. Genetically deleting SLC7A11 expression is not sufficient to induce ferroptosis in mice but promotes iron overload. Ferroptosis occurs in ion-induced SLC7A11^−/−^ cells, suggesting that the loss of SLC7A11 is favorable to the induction of ferroptosis, especially in high iron (Wang et al., 2017). Impaired cystine uptake and increased production of ROS contribute to the occurrence of ferroptosis, suggesting that SLC7A11 may prevent ferroptosis during iron overload.

#### 3.2.3 ATG6 Interacts With SLC7A11 and Inhibits its Activity

ATG6, also known as BECN1, is a core component of the phosphatidylinositol 3-kinase III (PI3K-III) complex. During cell autophagy, it plays a key role in promoting the formation of autophagosomes. Excessive autophagy can promote ferroptosis (Liu et al., 2020b; Zhou et al., 2020). Through binding to cytoplasmic high mobility group protein 1 (HMGB1), BECN1 stimulates autophagy-dependent ferroptosis. In addition to autophagy-induced initiation of ferroptosis, AMPK-mediated phosphorylation of ATG6 at Ser90/93/96 also triggers ferroptosis by inhibiting the activity of SLC7A11 (Adachi et al., 2008). ATG6 inhibits the activity of SLC7A11 by forming complexes by binding to the key component of the cystine/glutamate antiporter SLC7A11. Knockdown of ATG6 inhibits erastin-induced ferrotoxicity.

#### 3.2.4 ACSL4 is a Specific Biomarker and Driving Factor for Ferroptosis

ACSL4 is an enzyme involved in fatty acid metabolism and considered a specific biomarker and driving factor for ferroptosis. Overexpression of ACSL4 increases the levels of PUFAs in phospholipids, which are prone to oxidative stress and subsequent ferroptosis (Tang D. et al., 2021). Activation of ACSL4 is an important event in the enzymatic pathway to produce phospholipid hydroperoxide (Yang et al., 2016; Xie et al., 2017).

It has been reported that ACSL4 is expressed at low levels in the ferroptosis-resistant cell lines LNCaP and K562 compared with the ferroptosis-sensitive cell lines HepG2 and HL60. However, the expression levels of other ACSL proteins, such as ACSL1, ACSL3, ACSL5, and ACSL6, are irrelevant to sensitivity to ferroptosis (Yuan et al., 2016).

A genome-wide functional screen using the clustered regularly interspaced short palindromic repeat (CRISPR)-Cas9 system validated ACSL4 as an essential component of ferroptosis. Knockdown of ACSL4 suppressed erastin-induced sensitivity to ferroptosis in HepG2 and HL60 cells, while overexpression of ACSL4 reversed LNCaP and K562 cell susceptibility to ferroptosis. Furthermore, ACSL4-mediated production of 5-hydroxyeicosatetraenoic acid (5-HETE) promoted ferroptosis (Yuan et al., 2016). Co-silencing of GPX4 and ACSL4 resulted in a marked resistance to ferroptosis and an increase in omega-6 fatty acids on the cell membrane (Doll et al., 2017). It is concluded that ACSL4 is not only a specific biomarker of iron atrophy but also an important driver.

#### 3.2.5 Nrf2 Regulates Ferroptosis by Mediating Oxidative Stress

Nuclear factor E2–related factor 2 (Nrf2) is a stress-inducible transcription factor that is regulated by three E3-ubiquitin-ligase-complexes. The intracellular level of Nrf2 remains low under normal circumstances. Stimulated by an endogenous or exogenous factor, Nrf2 cannot be timely degraded and then translocated into the nucleus, where the transcription of antioxidant responsive element (ARE) initiates. Notably, a large number of proteins and enzymes inducing ferroptosis serve as targets of Nrf2. Nrf2 is of significance in iron metabolism. It controls the intracellular level of free iron by mediating storage proteins and SLC40A1, which is responsible for transporting free iron outside the cell, thus regulating ferroptosis (Gao et al., 2019b). Moreover, Nrf2 is able to regulate multiple glutathione synthetases and enzymes of glutathione metabolism (LaVaute et al., 2001; Arosio et al., 2017). GPX4 is also a transcriptional target of Nrf2 (Gao et al., 2016; Torii et al., 2016). Thus, Nrf2 is believed to be a key regulator of lipid peroxidation and ferroptosis (Dodson et al., 2019).

#### 3.2.6 NFS1 and ISCs Participate in Ferroptosis

Iron–sulfur clusters (ISCs) are synthesized from cysteine catalyzed by cysteine desulfurase (NFS1) (Alvarez et al., 2017). ISCs are redox-active protein cofactors that are present in at least 48 enzymes in mitochondria (Imlay, 2006; Stehling et al., 2014). NFS1 sensitizes cells to ferroptosis by activating the iron-starvation response by increasing transferrin receptor (TFRC) and decreasing ferritin heavy chain (FTH) levels. Co-inhibition of NFS1 and cysteine transport triggers ferroptosis. Deficiency of ISCs can also activate the iron-starvation response, which, along with the inhibition of glutathione biosynthesis, induces ferroptosis. Taken together, NFS1 and ISCs are involved in ferroptosis (Alvarez et al., 2017; Wu et al., 2020).

#### 3.2.7 Other Regulators of Ferroptosis

Novel regulators of ferroptosis are emerging with in-depth study, serving as promising therapeutic targets. Arachidonate 5-lipoxygenase (ALOX5) is an important enzyme that catalyzes lipid peroxidation reactions and plays an important role in ferroptosis (Mao et al., 2019). Pharmacological inhibition using zileuton-inhibited ferroptosis and exerted an indirect neuroprotective effect on glutamate-treated HT-22 cells (Liu et al., 2015). Consistent with this result, knockdown of ALOX5 protected neurons from ferroptosis in hemorrhagic stroke mice by neutralizing lipid peroxidation (Karuppagounder et al., 2018). This suggests that ALOX5 is an important regulator of ferroptosis (Sun Q. Y. et al., 2019).

Metallothionein-1G (MT1G) negatively regulates ferroptosis in human hepatocellular carcinoma (HCC) cells. Knockdown of MT1G significantly enhances end-product levels (e.g., MDA) of lipid peroxidation in cells treated with erastin and sorafenib, thus inducing ferroptosis by the Fenton reaction and the production of ROS. Nevertheless, knockdown of MT1G does not significantly influence the level of Fe^2+^ or iron metabolism genes such as FTH1, TFR1, and DMT1. These results indicate that MT1G inhibits ferroptosis by regulating lipid peroxidation without influencing the production and metabolism of Fe^2+^. Genetic and pharmacological inhibition of MT1G can promote sorafenib-induced ferroptosis by increasing GSH depletion–mediated lipid peroxidation. Therefore, MT1G is an important factor for ferroptosis (Sun et al., 2016).

Sirtuin 6 (SIRT6) also plays an important role in the regulation of ferroptosis. Sodium sulfide inhibits ferroptosis by upregulating SIRT6 in the prefrontal cortex of mice with diabetes mellitus (Wang et al., 2021). Knockdown of SIRT6 promotes ferroptosis in gastric cancer cells (Cai et al., 2021). Through the Nrf2 signaling pathway, SIRT6 is of great significance in oxidative stress and ferroptosis (Pan et al., 2016). Knockdown of SIRT6 increases the accumulation of ROS (Cai et al., 2021), while its overexpression reduces ROS levels in podocytes and thus alleviates oxidative stress (Fan et al., 2019). Inconsistently, it has been reported that SIRT6 stimulates the release of ROS in papillary thyroid cancer cells, which may be attributed to the heterogeneity of cell types (Yu et al., 2019). Therefore, more investigations are needed to clarify the biological function of SIRT6 in ferroptosis.

Multidrug resistance–associated protein 5 (MRP5, ABCC5) is a regulator of ferritin formation in HCC cells, which also participates in the progression of ferroptosis (Huang et al., 2021). ABCC5 inhibits ferroptosis by upregulating intracellular GSH and reducing the accumulation of lipid peroxidation by stabilizing the SLC7A11 protein. In contrast, knockdown of ABCC5 induces ferroptosis.

## 4 Ferroptosis and Drug Resistance

Drug resistance of cancer cells includes intrinsic and acquired resistance (Hayes and Wolf, 1990). It remains a huge challenge that significantly limits the efficacy of anticancer treatment, and great efforts have been made to overcome drug resistance. A growing amount of clinical evidence has shown that targeting ferroptosis may be a promising way to overcome drug resistance and enhance the therapeutic efficacy of anticancer treatment. Ferroptosis inducers are able to reverse the acquired resistance of cancer cells to lapatinib, cisplatin, docetaxel, sorafenib, etc. (Viswanathan et al., 2017). Inhibition of xCT and GPX4 can induce cancer cell death to conventional chemotherapy or radiotherapy (Xie et al., 2016). Inhibition of xCT enhances the sensitivity of cancer cells to anticancer agents by consuming GSH by blocking the uptake of cystine (Yoshikawa et al., 2013; Liu et al., 2017). A high-mesenchymal cell state would decrease the sensitivity of multiple types of cancer cells. It was found that the therapy-resistant high-mesenchymal cell state contributes to the escape from ferroptosis by regulating lipid peroxidation. Inhibition of GPX4 causes peroxide reactions mediated by intracellular iron, thus leading to ferroptosis. Therefore, induction of ferroptosis can effectively eliminate the high-mesenchymal cell state in cancer cells (Hangauer et al., 2017).

We summarized the mechanisms underlying ferroptosis in overcoming the drug resistance of currently approved anticancer agents. The role of ferroptosis in treatment resistance, strategies to overcome resistance in cancer, and strategies to enhance treatment efficacy by regulating ferroptosis in cancer are listed in Tables 1 and 2, respectively.

**TABLE 1:** Role of ferroptosis in drug resistance and the strategy to overcome treatment resistance in cancer.

Treatment strategy	Treatment	Cancer type	Strategy to overcome resistance	References
Chemotherapy	Cisplatin	Head and neck cancer	CDDP + erastin and sulfasalazine	Roh et al. (2016)
		Head and neck cancer	CDDP + Artesunate	Roh et al. (2017)
		Osteosarcoma	CDDP + Erastin and RSL3	Liu and Wang (2019)
		Ovarian cancer	CDDP + Erastin	Li et al. (2020)
		Gastric cancer	CDDP + Erastin	Haiyang Zhang et al. (2020)
		Non-small-cell lung cancer	CDDP + RSL3	Deng et al. (2021)
		Gastric cancer	CDDP + erastin RSL3 or antagonist liproxstatin-1	Dazhi Fu et al. (2021)
		Subcutaneous tumor	CDDP+11β-hydroxy-ent-16-kaurene-15-one	Yong Sun et al. (2021)
		Pancreatic adenocarcinoma	CDDP + gemcitabine	Wei et al. (2022)
		Ovarian cancer	CDDP + mTOR inhibitor	Hua-Wen Li et al. (2022)
		Triple-negative breast cancer	CDDP + HLF-knockdown	Hengyu Li et al. (2022)
	Oxaliplatin	Colorectal cancer	Oxaliplatin + RSL3	Changshun Yang et al. (2021)
		Colorectal cancer	Oxaliplatin + GPX4 inhibitor	Changshun Yang et al. (2021)
	Platinum	Non-small-cell lung cancer	Platinum + GPX4 inhibitor	Wenwen Liu et al. (2021)
Chemotherapy	Docetaxel	Ovarian cancer	Erastin + Docetaxel	Zhou et al. (2019)
	Paclitaxel	Uterine serous carcinoma	Paclitaxel + Sulfasalazine	Sugiyama et al. (2020)
	Gemcitabine	Pancreatic cancer	ARF6 abrogation	Zeng Ye et al. (2020)
		Pancreatic cancer	Gemcitabine + RSL3	Tang et al. (2020)
	5-fluorouracil	Colorectal cancer	Gemcitabine + Ferroptosis inducer	Chaudhary et al. (2021)
	Temozolomide	Glioblastoma	Temozolomide + ALZ003 upregulation	Xin Chen et al. (2020)
Targeted therapy	Sunitinib	Renal cell carcinoma	Sunitinib + Artesunate	Markowitsch et al. (2020)
	Sorafenib	Hepatocellular carcinoma	Sorafenib + Metallothionein (MT)-1G inhibition	Sun et al. (2016)
		Gastric cancer	Sorafenib + Sirtuins 6 inhibition	Cai et al. (2021)
		Hepatocellular carcinoma	Sorafenib + ABCC5 inhibition	Huang et al. (2021)
		Hepatocellular carcinoma	Sorafenib + Targeting YAP/TAZ or ATF4	Gao et al. (2021)
Targeted therapy	Gefitinib	Non-small-cell lung cancer	Gefitinib + GPX4 inhibition	Viswanathan et al. (2017)
	Cetuximab (anti-EGFR antibody)	Colorectal cancer	Cetuximab + Vitamin C	Lorenzato et al. (2020)
	EGFR-TKIs	EGFR-activating mutant lung adenocarcinoma	EGFR-TKIs + Vorinostat	Zhang et al. (2021)
	Androgen Receptor inhibitors	Prostate cancer	Androgen Receptor inhibitors +2,4-dienoyl-CoA reductase (DECR1)	Blomme et al. (2020)
		Prostate cancer	Androgen Receptor inhibitors + GPX4 inhibition	Tousignant et al. (2020)
		Prostate cancer	Androgen Receptor inhibitors +2,4-dienoyl-CoA reductase (DECR1)	Nassar et al. (2020)
	BRAF inhibitor	Melanoma	BRAF inhibition + Target SREBF2	Hong et al. (2021)
	Vemurafenib	Melanoma	Vemurafenib + Ferroptosis-inducing drugs	Tsoi et al. (2018)
	Abemaciclib, Sorafenib	Hepatocellular carcinoma	Sorafenib + PSTK inhibition	Chen et al. (2022)
	Lapatinib	Non-small-cell lung cancer	Lapatinib + GPX4 inhibition	Ni et al. (2021)
Multi-drug	Multi-drug resistance	Drug-resistant Breast Cancer Cells	Basic therapy + Pt-AuNS prodrugs	Del Valle et al. (2020)
Multi-drug	Multi-drug resistance	Various sensitive and drug-resistant phenotypes	Standard treatment + Ardisiacrispin B	Mbaveng et al. (2018b)
		Various sensitive and drug-resistant cell lines	Basic therapy + Epunctanone	Mbaveng et al. (2018a)
		Various sensitive and drug-resistant cell lines (leukemia cells)	Basic therapy + Ungeremine	Mbaveng et al. (2019)
Multiple drugs	Lapatinib, Erlotinib	Breast cancer, Non-small-cell lung cancer, ovarian cancer, melanoma	Standard treatment + GPX4 inhibition	Hangauer et al. (2017)
	Carboplatin, Paclitaxel, Vemurafenib, Dabrafenib, Trametinib, Dabrafenib			
Immunotherapy	Anti-PD-1/PD-L1 blockade	Mammary carcinoma	Anti-PD-1/PD-L1 blockade + TYRO3 inhibition	Zhou Jiang et al. (2021)
		Bladder cancer	Anti-PD-1/PD-L1 blockade + ACSL4activation	Liao et al. (2022)
Radiotherapy	Radiation	Clinically relevant radioresistant (CRR) cells	Basic therapy + miR-7-5p knockdown	Tomita et al. (2021)
		Lung cancer, fibrosarcoma cell, breast adenocarcinoma cell	Ionizing radiation + Ferroptosis inducers	Lei et al. (2020)

**TABLE 2:** Strategy to enhance therapeutic efficacy of approved treatment through regulating ferroptosis in cancer.

Treatment strategy	Treatment	Cancer type	Combination strategy to enhance treatment efficacy	References
Chemotherapy	Cisplatin	Ovarian cancer	CDDP + Erastin	Cheng et al. (2021)
		PDAC	CDDP + DHA	Du et al. (2021)
		Osteosarcoma	CDDP + Ursolic Acid	Zhen Tang et al. (2021)
	Gemcitabine	Pancreatic cancer	Chrysin + Gemcitabine	Zhou et al. (2021)
	Doxorubicin	Osteosarcoma	Doxorubicin + Ferrate	Jingke Fu et al. (2021)
		Ovarian cancer	Doxorubicin + RSL3	Gao et al. (2019a)
Targeted therapy	Cetuximab	KRAS mutant colorectal cancer	Cetuximab + RSL3	Jiawen Yang et al. (2021)
	Gefitinib	Triple negative breast cancer	Gefitinib + GPX4 Inhibition	Song et al. (2020)
	Everolimus	Renal cell carcinoma	Everolimus + Erastin/RSL3	Yangyun et al. (2022)
	Sorafenib	HepG2 cell (hepatoellular carcinomas)	Dihydroartemisinin (DHA)+Sorafenib	Cui et al. (2022)
Immunotherapy	PD-L1 blockade	Ovarian cancer	PD-L1 blockade + cyst(e)inase	Wang et al. (2019)
		Multiple cancer types	PD-L1 blockade + Low-dose arachidonic acid	Liao et al. (2022)
Radiotherapy	Radiation	Lung adenocarcinoma, glioma	Radiation + Ferroptosis Inducers	Zeng Ye et al. (2020)
		Melanoma	Ionizing radiation + ACSL3 KO/cyst(e)inase/SLC7A11 KO	Lang et al. (2019)

### 4.1 Chemotherapy

#### 4.1.1 Platinum Drugs

Cisplatin, also known as DDP, is a classic anticancer drug that is the most widely used in clinical application. It can be applied to many types of solid tumors, such as bladder cancer, ovarian cancer, testicular cancer, lung cancer, gastric cancer, colorectal cancer, and head and neck cancer (HNC) (Galluzzi et al., 2014). Preclinical studies have shown that inhibition of xCT reverses the resistance of HNC cells to cisplatin by inducing ferroptosis (Roh et al., 2016). The antimalarial drug artesunate has been repurposed as an anticancer drug, although its sensitivity in cisplatin-resistant HNC cells is relatively low. Inactivation of the Nrf2-ARE pathway increases the sensitivity of drug-resistant HNC cells to artesunate and reverses the resistance of drug-resistant cells to ferroptosis (Roh et al., 2017). Cisplatin-resistant osteosarcoma cells present inhibited ferroptosis after exposure to low-dose cisplatin, which is reactivated by the induction of ferroptosis inducers (Liu and Wang, 2019). Cotreatment with cisplatin and ferroptosis inducers (erastin and RSL3) significantly increases the sensitivity of drug-resistant cells to cisplatin. In gastric cancer cells, cisplatin and paclitaxel upregulate miR-522 and then downregulate ALOX15 in cancer-associated fibroblasts, thereafter leading to acquired chemoresistance by inhibiting the accumulation of lipid ROS and ferroptosis (Zhang H. et al., 2020). Therefore, the induction of ferroptosis is favorable to inhibit the drug resistance of cisplatin and paclitaxel. ATF3 (activating transcription factor 3) may induce ferroptosis by blocking the Nrf2/Keap1/xCT signaling pathway and reverse the sensitivity of gastric cancer cells to cisplatin (Fu D. et al., 2021). Ent-Kaurane derivatives are promising in chemotherapy and are capable of reversing the resistance to cisplatin by dual inhibition of PRDX I/II and GSH (Sun Y. et al., 2021). Non-small-cell lung cancer (NSCLC) accounts for approximately 85% of lung cancer cases (Ferlay et al., 2015). Multicourse cisplatin–based chemotherapy is a standard adjuvant therapy for NSCLC, although its clinical benefits are limited by drug resistance. Cisplatin induces activation of the Nrf2/xCT pathway in different NSCLC cell lines, and the degree of activation is correlated with the resistance level to cisplatin. Nrf2 and xCT are significantly upregulated in cisplatin-resistant NSCLC cells. The classic ferroptosis inducers erastin and sorafenib significantly induce ferroptosis in cisplatin-resistant NSCLC cells. Interestingly, low-dose cisplatin induction combined with erastin/sorafenib effectively inhibits the *in vitro* growth of cisplatin-resistant NSCLC cells, indicating that erastin/sorafenib-induced ferroptosis may provide a novel option for combating cisplatin-resistant NSCLC (Li et al., 2020). It has been reported that the Wnt/Nrf2/GPX4 signaling pathway promotes acquired chemoresistance by inhibiting ferroptosis and highly consuming GSH.

GPX4 inhibitors can enhance the therapeutic effect of platinum-based drugs on drug-resistant lung cancer brain (Liu W. et al., 2021). Erastin induction also enhances the therapeutic effect of cisplatin on ovarian cancer through ROS-mediated ferroptosis, serving as a novel strategy for overcoming cisplatin resistance (Cheng et al., 2021). The bioactive component ursolic acid isolated from kiwifruit possesses a strong anticancer effect on osteosarcoma cells, the combination of which and cisplatin further presents a synergistic effect on killing osteosarcoma cells. Consistently, low-dose cisplatin combined with ursolic acid significantly inhibited the malignant growth of osteosarcoma in an *in vivo* xenograft model through ferroptosis caused by the degradation of ferritin and accumulation of intracellular ferrous ions. Moreover, ursolic acidursolic acid enhances cisplatin-induced DNA damage in osteosarcoma cells. It is suggested that ursolic acid is a nontoxic adjuvant that enhances the chemotherapeutic effect of osteosarcoma (Tang Z. et al., 2021).

Oxaliplatin can prolong the median disease-free survival (DFS) and overall survival (OS) in patients with advanced colorectal cancer (CRC). However, less than 40% of CRC patients can benefit from oxaliplatin due to drug resistance (Sun et al., 2017a; Sun F. et al., 2019). With advanced research on the pathogenesis and drug resistance of CRC, several molecular mechanisms underlying the high rate of resistance have been identified. For example, activation of ABC transporters and hypermethylation of CpG islands are involved in oxaliplatin resistance (De Mattia et al., 2015; Sun et al., 2017b). Unfortunately, the combination therapy of oxaliplatin and other drugs does not achieve a satisfactory outcome, and long-term application even aggravates adverse events. It has been reported that GPX4 is more highly expressed in advanced CRC specimens than in paracancerous (Méplan et al., 2010). High-dose RSL3 treatment induces ferroptosis in CRC cells by stimulating the production of lipid peroxides by downregulating GPX4 (Sui et al., 2018). It is speculated that high levels of GPX4 may induce the resistance of CRC to oxaliplatin, and its combination with ferroptosis inducers is expected to overcome drug resistance (Yang C. et al., 2021).

Taken together, ferroptosis is involved in the resistance of multiple types of cancers to platinum-based drugs, and targeting ferroptosis is a promising strategy to overcome resistance to cisplatin and oxaliplatin.

#### 4.1.2 Docetaxel and Paclitaxel

Docetaxel is a derivative of paclitaxel that has been widely used in the treatment of ovarian cancer, especially as a first-line chemotherapy alternative to paclitaxel. It can be used alone or in combination with other chemotherapeutic drugs, such as paclitaxel, which arrests cell cycle progression by inhibiting microtubule growth (Li et al., 2019; Lu and Meng, 2019). Although docetaxel has a remarkable anticancer effect, drug resistance to it remains a major challenge in clinical application. Serving as a ferroptosis inducer, low-level erastin is able to strongly downregulate SLC7A11 (Sato et al., 2018), thus preventing the transport of cystine and leading to the depletion of GSH (Dixon et al., 2012; Yu Y. et al., 2017). A preclinical study has found that erastin can reverse ABCB1-mediated resistance to docetaxel in ovarian cancer, indicating that the combination of erastin and docetaxel is a promising strategy available to chemotherapy-resistant patients with ovarian cancer (Zhou et al., 2019). The therapeutic potential of the xCT inhibitor sulfasalazine (SAS) has been identified in a paclitaxel-resistant uterine serous carcinoma cell line. Compared with sensitive cells, SAS is more cytotoxic to paclitaxel-resistant cells by inducing ferroptosis rather than apoptosis. It is indicated that xCT inhibitors may be effective for patients with relapsed paclitaxel-resistant uterine serous carcinoma (Sugiyama et al., 2020).

#### 4.1.3 Gemcitabine

Gemcitabine is the basic chemotherapy drug for pancreatic ductal adenocarcinoma (PDAC), the combination of which and other drugs, such as cisplatin, has become the most widely applied therapeutic strategy for PDAC (Heinemann, 2001; Tadros et al., 2017). However, the acquired resistance of gemcitabine (Binenbaum et al., 2015) and cisplatin (Galluzzi et al., 2012) leads to treatment failure. It is urgent to overcome chemotherapy resistance, thus enhancing the therapeutic efficacy of PDAC. ARF6 does not directly regulate lipid peroxidation, but it sensitizes PDAC cells to oxidative stress, especially RSL3-induced lipid peroxidation. ARF6 also regulates gemcitabine resistance by downregulating DCK and hENT1 (Ye Z. et al., 2020). Through analyzing the correlation between ferroptosis-related genes (FRGs) and the sensitivity of anticancer drugs using Lasso penalized Cox regression analysis, it was found that spermidine/spermine N1-acetyltransferase 1 (SAT1) significantly influences resistance to cisplatin and gemcitabine. *In vitro* data revealed that gemcitabine combined with cisplatin can induce ferroptosis in AsPC1 cells by upregulating SAT1 (Wei et al., 2022). RNA sequencing in 31 types of cancer specimens showed that 14/31 are highly sensitive to ferroptosis inducers. Serving as the main target of ferroptosis, xCT is upregulated in gemcitabine-resistant PDAC cells (Tang et al., 2020). The combination of immunotherapy and ferroptosis inducers is considered a promising option for the treatment of PDAC. The sensitivity to ferroptosis in PDAC patients has been found to be correlated with the high infiltration of CD8^+^ T cells, type II interferon responses, and immune checkpoints. Human carbonyl reductase 1 (CBR1) protects cells from oxidative stress. Through the immunohistochemical staining of pancreatic cancer (PCA) samples in the GEPIA database, CBR1 was found to be upregulated in PCA and significantly correlated with the clinical characteristics of PCA patients. Knockdown of CBR1 inhibits the proliferation of PCA cells by regulating the production of ROS. Moreover, knockdown of CBR1 contributes to enhancing the sensitivity of PDAC cells to gemcitabine. The flavonoid chrysin can directly bind to CBR1, which inhibits its enzymatic activity at the molecular and cellular levels, thereby increasing ROS levels and ROS-dependent autophagy. It induces ferroptosis in PCA cells by degrading ferritin heavy polypeptide 1 (FTH1) and enhancing the intracellular free iron levels, which ultimately increases the sensitivity to gemcitabine (Zhou et al., 2021).

#### 4.1.4 5-Fluorouracil

Surgery is the first-line treatment for CRC, and postoperative adjuvant chemotherapy of 5-fluorouracil (5-FU) and oxaliplatin is applied to patients with stage III and IV CRC. However, drug resistance develops in most CRC patients. Lipocalin 2 is a secreted glycoprotein that regulates iron homeostasis (Playford et al., 2006). It is upregulated in many types of tumors, although the oncogenic mechanism remains unclear. Overexpression of Lipocalin 2 leads to the resistance of CRC to 5-FU by inhibiting ferroptosis *in vitro* and *in vivo*, which is attributed to the reduction in intracellular iron levels and upregulation of GPX4 and xCT. The Lipocalin 2 monoclonal antibody is capable of suppressing chemotherapy resistance and transformation in xenograft mice. Moreover, the expression level of Lipocalin 2 is positively correlated with that of xCT in human CRC specimens. Lipocalin 2 is a potential therapeutic target for overcoming 5-FU resistance by regulating ferroptosis (Chaudhary et al., 2021).

#### 4.1.5 Temozolomide and Doxorubicin

Temozolomide (TMZ) is a methylated antitumor triazene compound that induces apoptotic and autophagic cell death through postreplicative mismatch repair (D'Atri et al., 1998). However, at least 50% of patients treated with TMZ do not respond to TMZ. Therefore, increasing the efficacy of TMZ is of great important in cancer treatment (Lee, 2016). The expression of xCT is closely related to the malignancy of brain tumors. The activity of temozolomide on glioma cells was shown to be dependent on the expression of xCT and could be promoted through ferroptosis (Sehm et al., 2016). Erastin and sorafenib are partial xCT inhibitor that induces ferroptosis in a variety of tumor cells. Glioma cells overexpressing xCT tolerated erastin and sorafenib-induced cell death in a concentration-dependent manner, whereas knockdown of xCT increased the toxicity of erastin and sorafenib to glioma cells. More importantly, the combined use of erastin and TMZ enhanced the efficacy, suggesting that combination with ferroptosis inducers is an effective strategy to enhance the efficacy of the first-line treatment agent TMZ (Sehm et al., 2016).

Doxorubicin is a common chemotherapy drug used to treat many cancer types, including breast cancer, bladder cancer, Kaposi’s sarcoma, lymphoma, and acute lymphocytic leukemia (Carvalho et al., 2009). Doxorubicin has poor activity against drug-tolerant drug-tolerant persister cancer cells (PCCs). This may be related to EMT. Targeting the ferroptosis pathway has high activity to eliminate cells in the EMT state. A study in a Doxorubicin (Dox)-resistant human ovarian cancer model found that RSL3 encapsulated in the polymer micelles was able to induce ferroptosis in PCCs by targeting GPX4, thereby overcoming resistance to doxorubicin. Following the rapid release of cargo upon initiation of free radicals in the tumor microenvironment, RSL3-loaded micelles induced lipid peroxidation and decreased intracellular glutathione level, which in turn decreased CD133^+^ and aldehyde dehydrogenase (ALDH^+^) PCCs population (Gao et al., 2019a). Hypoxic microenvironment promotes cancer resistance to chemotherapy. A recent study found that targeting ferroptosis can enhance the therapeutic effect of doxorubicin in hypoxic osteosarcoma by activating ferroptosis, showing great potential to overcome hypoxia-induced drug resistance. The authors integrated ferrate and doxorubicin into biocompatible hollow mesoporous silica nanoplatforms. When the system was activated with ultrasound, ferrate and doxorubicin were released together. The released ferrate efficiently reacts with water as well as hydrogen peroxide and glutathione in tumor cells for TME-independent reoxygenation and glutathione depletion. Reoxygenation downregulates the expression of HIF 1α and P-glycoprotein in tumor cells, sensitizing the anticancer effects of doxorubicin. Furthermore, glutathione depletion inactivated GPX4, which inhibits lipid peroxides, and enhanced ferroptosis, demonstrating the potential to overcome drug resistance by inducing sensitized apoptosis and collaborative ferroptosis of tumor cells (Fu J. et al., 2021).

### 4.2 Targeted Therapy

#### 4.2.1 Sorafenib

Sorafenib is an inhibitor of multiple oncogenic kinases, which has been approved for the treatment of advanced renal cell carcinoma (Escudier et al., 2007). It is also the only systemic therapy approved for patients with advanced HCC who cannot be operated (Siegel et al., 2010). The therapeutic efficacy of sorafenib on multiple types of solid tumors has been validated (Meyer et al., 2014; Mammatas et al., 2020). However, drug resistance to sorafenib results in the poor prognosis of HCC (Llovet et al., 2008; Cheng et al., 2009). Expanded studies have shown that targeting ferroptosis is an effective method to overcome sorafenib resistance.

MT-1G is a negative regulator of ferroptosis in human HCC cells, which is upregulated in drug-resistant cancer cells and considered the cause of acquired resistance (Bahnson et al., 1991). MT-1G is a key regulator of sorafenib resistance. Metallothioneins (MTs) have a high affinity for divalent heavy metal ions, which are important to prevent heavy metals and oxidative injury. Sorafenib targets the mRNA and protein levels of MT-1G by activating the transcription factor Nrf2. MT-1G inhibits ferroptosis by regulating lipid peroxidation rather than the production and metabolism of Fe^2+^. The genetic and pharmacological inhibition of MT-1G promotes ferroptosis in sorafenib-resistant cells and enhances the anticancer activity of sorafenib both *in vitro* and *in vivo*. Therefore, regulating MT-1G and targeting ferroptosis are expected to effectively reverse acquired resistance to sorafenib (Sun et al., 2016).

SIRT6, a member of the sirtuin family, is an NAD+-dependent enzyme essential for various biological functions (Michishita et al., 2005). SIRT6 has been reported to be upregulated in sorafenib-resistant GC cells and inhibits ferroptosis by upregulating GPX4 and activating the Keap1/Nrf2 signaling pathway (Cai et al., 2021). Targeting the SIRT6/Keap1/Nrf2/GPX4 signaling pathway facilitates ferroptosis in cancer cells, and this property may be may be one of the potential strategies to address the resistance of cancer cells to sorafenib.

ABCC5 induces acquired resistance to sorafenib *in vitro* by inhibiting ferroptosis. It is upregulated in sorafenib-resistant HCC cells, and knockdown of ABCC5 significantly reverses the sensitivity to sorafenib (Huang et al., 2021). As a result, ABCC5 is a regulator of ferroptosis that may be useful to overcome the acquired resistance of HCC to sorafenib.

YAP/TAZ also plays a key role in the resistance of sorafenib to HCC. It induces the expression of SLC7A11 in a TEAD-dependent manner and maintains the homeostasis of intracellular GSH, thereby suppressing sorafenib-induced ferroptosis in HCC cells. Inhibition of the antioxidant pathway regulated by YAP/TAZ and ATF4 may resensitize drug-resistant HCC to sorafenib (Chen et al., 2022). In addition, dihydroartemisinin (DHA) can enhance the anticancer effect of sorafenib by downregulating GSH-related proteins in the iron metabolism pathway, thereby enhancing the function of sorafenib in inducing ferroptosis in HepG2 cells (Cui et al., 2022).

#### 4.2.2 EGFR Inhibitor

##### 4.2.2.1 EGFR-Tyrosine Kinase Inhibitor

Epidermal growth factor receptor (EGFR) is the most common mutation driving the carcinogenesis of lung adenocarcinoma (LUAD), with a mutation rate of up to 55% in Asian LUAD patients (Zhang et al., 2021). Serving as the first-line treatment of EGFR-mutant LUAD, acquired resistance to EGFR-tyrosine kinase inhibitors (EGFR-TKIs) remarkably reduces therapeutic efficacy. Moreover, approximately 20–30% of EGFR-mutant LUAD patients possess intrinsic resistance to EGFR-TKIs (Wang et al., 2016). It has been reported that cells with intrinsic or acquired resistance to EGFR-TKIs exhibit higher responses to ferroptosis inducers than EGFR-TKI-sensitive cells. The histone deacetylase (HDAC) inhibitor vorinostat promotes ferroptosis through downregulating xCT, resulting in a dramatic increase in hydroperoxides in EGFR-mutant lung cancer cells (Zhang et al., 2021). It is favorable to overcome the resistance of lung cancer cells to first-, second- and third-generation EGFR-TKIs (Zang et al., 2020).

Gefitinib is an oral EGFR TKI for the treatment of advanced NSCLC (Yang et al., 2017). However, acquired therapeutic resistance to gefitinib inevitably develops. Compared with parental HCC4006 cells, those with a high mesenchymal cell state and gefinitib resistance are highly sensitive to the inhibition of GPX4 (Ware et al., 2013). The loss of function of GPX4 induces ferroptosis in mesenchymal-state cells rather than epithelial-state cells (Viswanathan et al., 2017).

Drug resistance remarkably limits the application of gefitinib in triple-negative breast cancer (TNBC). TNBC cells are sensitive to erastin-induced ferroptosis (Yu H. et al., 2017). Later, expanded studies have shown that GPX4 negatively regulates ferroptosis in gefitinib-resistant TNBC cells, contributing to enhancing the anticancer effect of gefitinib (Song et al., 2020).

##### 4.2.2.2 Cetuximab

The long-term efficacy of the EGFR-targeting antibody cetuximab in advanced CRC patients is limited by the emergence of drug resistance (Diaz et al., 2012). High-dose vitamin C has the potential to induce ferroptosis, serving as a prooxidant therapeutic agent to fight against EGFR-resistant cancers (Chen et al., 2007). Cetuximab-resistant CRC cells are easily influenced by vitamin C-induced oxidative stress by altering the homeostasis of iron. The combination of cetuximab and vitamin C delays the emergence of drug resistance, which is a promising approach to alleviate cetuximab resistance in CRC.

The small molecule RSL3 is able to kill Ras-mutant cancer cells (Yang and Stockwell, 2008) and activate ferroptosis in them (Chen et al., 2021a). As a potent ferroptosis inducer, it promotes ferroptosis in cancer cells by directly inhibiting GPX4 (Shintoku et al., 2017). Therefore, induction of ferroptosis may be an effective strategy for the treatment of KRAS-mutant CRC. The combination of RSL3 and cetuximab synergistically stimulates the death of the KRAS-mutant CRC cell lines HCT116 and DLD-1. Cetuximab promotes lipid peroxidation and thereafter enhances RSL3-induced ferroptosis by targeting the Nrf2/HO-1 axis by activating the p38 mitogen-activated protein kinase (Yang J. et al., 2021). It is believed that the combination of RSL3 and cetuximab is favorable for the therapeutic efficacy of inducing ferroptosis in KRAS-mutant CRC.

#### 4.2.3 Androgen Receptor Inhibitors

In developed countries, Prostate cancer (PC) is the most common male cancer (Blomme et al., 2020). Many PC cases are androgen-sensitive and require the androgen receptor (AR) signaling pathway. Therefore, despite the fact that androgen deprivation therapy (ADT) leads to a high recurrence rate and may even exacerbate fatal castration-resistant prostate cancer (CRPC), it has long been considered the standard of care for advanced PC.

Selective AR inhibitors (ARIs) have shown promising therapeutic efficacy on PC, which significantly improve the clinical outcomes (Watson et al., 2015). The first-generation AR antagonist bicalutamide and the subsequent AR antagonists enzalutamide and apalutamide have achieved acceptable clinical benefits. Reactivation of the AR signaling pathway is a major driver of CRPC progression, which is responsible for mediating the metabolism of PC cells (Massie et al., 2011). It has been reported that persisting PC cells are GPX4-dependent and present hypersensitivity to ferroptosis, which is closely linked with lipid remodeling, increased lipid uptake and PUFA enrichment of membrane lipids. Furthermore, the activities of lipase and fatty acid desaturase are essential for GPX4-dependent development of persister cells (Tousignant et al., 2020).

Fatty acid β-oxidation (FAO) is a major bioenergy metabolism pathway in PC, serving as a promising therapeutic vulnerability. *In vitro* experiments demonstrated the therapeutic effect of targeting FAO on PC. 2,4-dienoyl-CoA reductase (DECR1) is a rate-limiting enzyme for the oxidation of PUFAs. It is also a negative regulator of AR-targeted genes, which stimulates the resistance of PC cells to AR-targeted therapy. DECR1 is significantly upregulated in PC tissues, the high level of which is correlated with poor recurrence-free survival of PC. Knockdown of DECR1 selectively suppresses the β-oxidation of PUFAs and the proliferation and migration of PC cells. Moreover, knockdown of DECR1 leads to the intracellular accumulation of PUFAs, which further triggers mitochondrial oxidative stress, lipid peroxidation and ferroptosis. Therefore, DECR1-mediated oxidation of PUFAs is a therapeutic target for overcoming drug resistance in PC (Nassar et al., 2020).

An activated AR signaling pathway is detected in ARI-resistant cells, and the presence of ARI resistance is linked to cell metabolism. Proteomic and metabolomic analyses have shown remarkable changes in glucose and lipid metabolism in ARI-resistant cells, in which the AR signaling pathway drives metabolic reprogramming. DECR1 is able to maintain lipid homeostasis in CRPC cells, and its deficiency causes ER stress and sensitizes CRPC cells to ferroptosis by enhancing PUFA levels. Knockdown of DECR1 impairs lipid metabolism and inhibits the growth of CRPC *in vivo* (Blomme et al., 2020). In conclusion, DECR1 plays a key role in the development of drug resistance in CRPC, serving as a vital therapeutic target.

#### 4.2.4 BRAF Inhibitors

**FIGURE 2:**
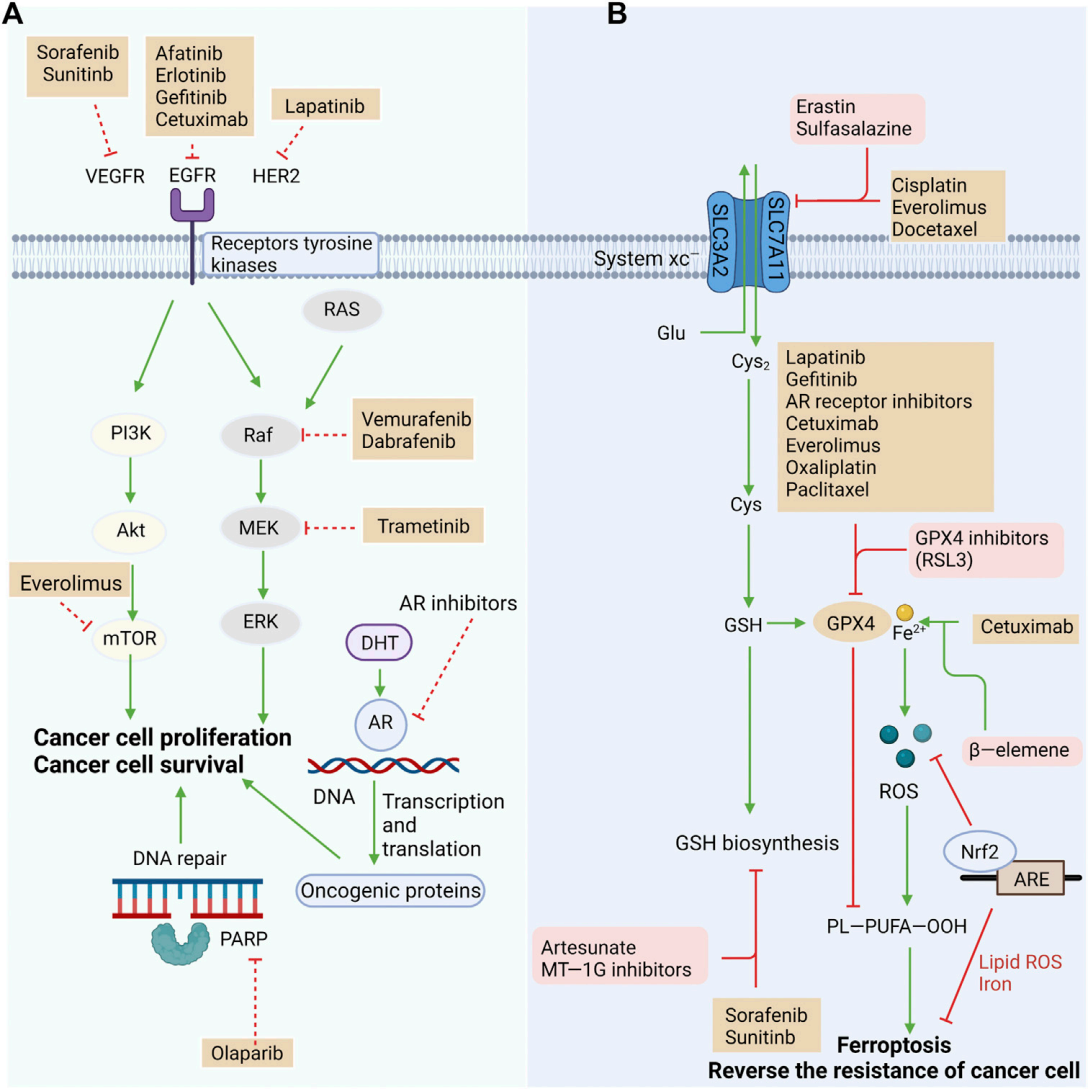
Reversing resistance or enhancing the efficacy of targeted therapy and chemotherapy by targeting the ferroptosis pathway. **(A)** Targeted drugs exert antitumor effects by blocking oncogenic signaling pathways, but innate or acquired resistance reduces their efficacy. **(B)** One of the mechanisms of resistance is reduced susceptibility to ferroptosis. Targeting multiple pathways in ferroptosis to restore their response to ferroptosis could eliminate resistance or improve the efficacy of existing standard treatments, including chemotherapy and targeted therapy. System Xc^−^ and GPX4 have critical roles in preventing ferroptosis and potential targets to reverse treatment resistance. Other factors that regulate the redox of intracellular lipid are also have critical roles in anticancer treatment resistance. Many approved drugs target those potential targets and may reverse the resistance by exploiting ferroptosis pathway.

Mutations in the oncogene v-raf murine viral oncogene homolog B1 (BRAF) are detected in many types of tumors. BRAF inhibitors have been approved for the treatment of melanoma (Davies et al., 2002). It has been reported that the combination of BRAF and MEK inhibitors rapidly shrinks BRAF V600E-mutated melanoma, although almost all cases suffer recurrence due to drug resistance (Hong et al., 2021). Melanoma cells are strongly invasive and present strong resistance to clinical interventions, except for immunotherapy and BRAF-targeted therapy. A recent study demonstrated that primary melanoma cells are affected by ROS, while their subsets are resistant to ROS and keep alive in the circulatory system highly enriched with oxygen (Piskounova et al., 2015). Circulating tumor cells (CTCs) in melanoma patients synergistically activate adipogenesis and ion homeostasis, resulting in intrinsic and acquired resistance to BRAF inhibitors. Furthermore, sterol regulatory element-binding protein (SREBP)-induced adipogenesis is significantly upregulated in in vitro cultured CTCs. It contributes to reducing intracellular iron pools, ROS levels, lipid peroxidation and ferroptosis by inducing the transcription of the iron carrier transferrin (TF). Vemurafenib induction upregulates endogenous SREBP in in vitro cultured CTCs, and knockdown of TF suppresses tumor formation by melanoma CTCs. Therefore, targeting SREBP may be a potential therapeutic strategy to inhibit resistance and metastasis of melanoma (Hong et al., 2021). The effect of reversing drug resistance or enhancing targeted therapy and chemotherapy through targeted ferroptosis is shown in Figure 2.

### 4.3 Radiotherapy

**FIGURE 3:**
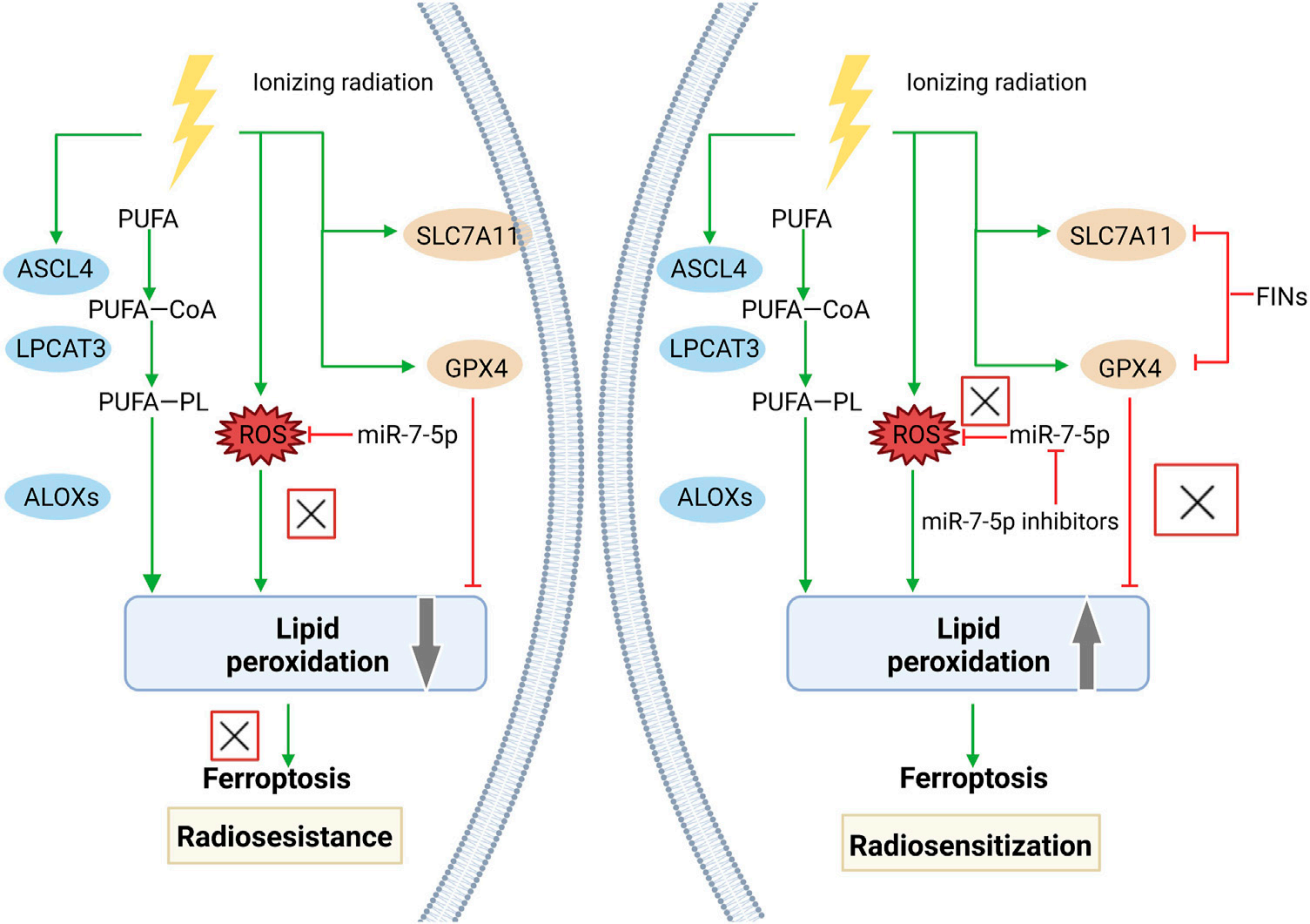
Reversal of radioresistance by targeting ferroptosis. Radioresistance remains a major factor in radiotherapy failure. Radiation therapy can lead to the production of massive ROS and upregulate the expression of ACSL4, promote lipid peroxidation and eventually cause ferroptosis. However, radiotherapy also induced an adaptive response in tumor cells. The expression of ferroptosis suppressors, including SLC7A11 and GPX4, was also significantly upregulated, which promoted cancer cell survival and radioresistance after radiotherapy. FINs that inhibit SLC7A11 or GPX4 can enhance the sensitivity of radioresistant cancer cells to IR-induced ferroptosis and reverse radioresistance. miR-7-5p controls radioresistance via ROS generation that leads to ferroptosis. Knockdown of miR-7-5p increased ROS and reversed radioresistance.

Radiotherapy is a vital option for cancer patients (Delaney et al., 2005), which breaks double strands in DNA through high-energy ionizing radiation (IR). The degree of DNA damage and the ability to repair DNA are the most critical factors in determining the death of inherent tumor cells from IR (Morgan and Lawrence, 2015). However, various types of cancers develop resistance to radiotherapy (Gerszten et al., 2009; Tang et al., 2017), which are associated with the activation of DNA repair and inhibition of cell apoptosis (Willers et al., 2013; Kim et al., 2015). In addition, targeting ferroptosis has emerged as a novel strategy to overcome radiotherapy resistance (Ye L. F. et al., 2020). Small molecules that activate ferroptosis by inhibiting xCT or GPX4 exert a synergistic effect on anticancer treatment alongside radiotherapy, in which DNA damage is not aggravated. Ferroptosis inducers contribute to expand the therapeutic efficacy of radiotherapy in a murine xenograft model and human patient-derived models of lung adenocarcinoma and glioma, suggesting that ferroptosis inducers may be potent radiosensitizers to expand the indication of radiotherapy (Ye L. F. et al., 2020). In a melanoma mouse model, knockout of ferroptosis suppressor ACSL3/SLC7A11 or using cyst(e)inase was found to significantly enhance the anticancer effect of radiotherapy (8 Gy, single fraction) by promoting tumor lipid oxidation and ferroptosis. Furthermore, immunotherapy sensitizes tumors to radiotherapy by promoting tumor-cell ferroptosis through IFNγ-induced SLC7A11 suppression, suggesting that ferroptosis may serve as a determinant of synergy between radiotherapy and immunotherapy (Lang et al., 2019). Clinically relevant radioresistant (CRR) cells are resistant to anticancer agents and H_2_O_2_, in which miR-7-5p is upregulated. Knockdown of miR-7-5p downregulates iron storage genes and upregulates the ferroptosis marker ALOX12, thus enhancing ROS levels and lipid peroxidation in CRC cells. Therefore, knockdown of miR-7-5p leads to increased sensitivity of CRR cells to ferroptosis, which is favorable to overcome resistance to radiotherapy (Tomita et al., 2021). IR induces ROS production and activates the ferroptosis marker ACSL4, which stimulates lipid peroxidation and ferroptosis. However, as an adaptive response of cells, IR also activates ferroptosis inhibitors such as SLC7A11 and GPX4, which may contribute to resistance to radiotherapy. Inactivation of SLC7A11 or GPX4 with ferroptosis inducers is able to sensitize radioresistant cancer cells and xenograft tumors to IR. Collectively, the combination of radiotherapy and ferroptosis inducers may synergistically produce a more pronounced anticancer efficacy (Lei et al., 2020). The mechanism by which radioresistance is reversed by targeting ferroptosis is shown in Figure 3.

### 4.4 Immunotherapy

Immune checkpoint blockade (ICB) therapy has been validated for its acceptable clinical efficacy on many types of cancers. However, only a few patients are responsive to ICB therapy, and its clinical benefits are largely limited by innate and acquired resistance. Ferroptosis is involved in resistance to ICB therapy. In a syngeneic mouse tumor model and patients receiving anti-PD-1/PD-L1 therapy, tumors expressing a high level of tyrosine-protein kinase receptor (Tyro3) present resistance to anti-PD-1/PD-L1 therapy by inhibiting ferroptosis (Jiang Z. et al., 2021). Tyro3 upregulates genes that inhibit ferroptosis (e.g., SLC40A1, SLC7A11, SLC3A2, GPX4, FTH1, BLVRB) but downregulates those that promote ferroptosis (e.g., SLC5A1, TFRC). Moreover, Tyro3 also contributes to the generation of a microenvironment that is favorable to tumor growth by reducing the ratio of M1/M2 macrophages. The combination of anti-PD-1 antibodies with Tyro3 inhibitors can reverse the responsiveness of resistant tumor cells by stimulating ferroptosis. Therefore, Tyro3 serves as a promising biomarker for predicting the efficacy of ICB therapy and overcoming resistance. IFN-γ released by CD8^+^ T cells downregulates SLC3A2 and SLC7A11, thereby inhibiting cystine uptake and promoting lipid peroxidation and ferroptosis. Induction of an engineered enzyme that degrades both cystine and cysteine enhances the immunity of ICB and ferroptosis in a mouse tumor model. In human melanoma tissues, both SLC7A11 and LC3A2 are negatively correlated with the number of CD8^+^ T cells, the expression level of IFN-γ and patient outcome. Those expressing a low level of SLC3A2 present a better responsiveness to nivolumab therapy. It is indicated that metabolic changes induced by cytotoxic T cells affect ferroptosis, and targeting this pathway is a potential therapeutic strategy to enhance the efficacy of ICB (Wang et al., 2019).

**FIGURE 4:**
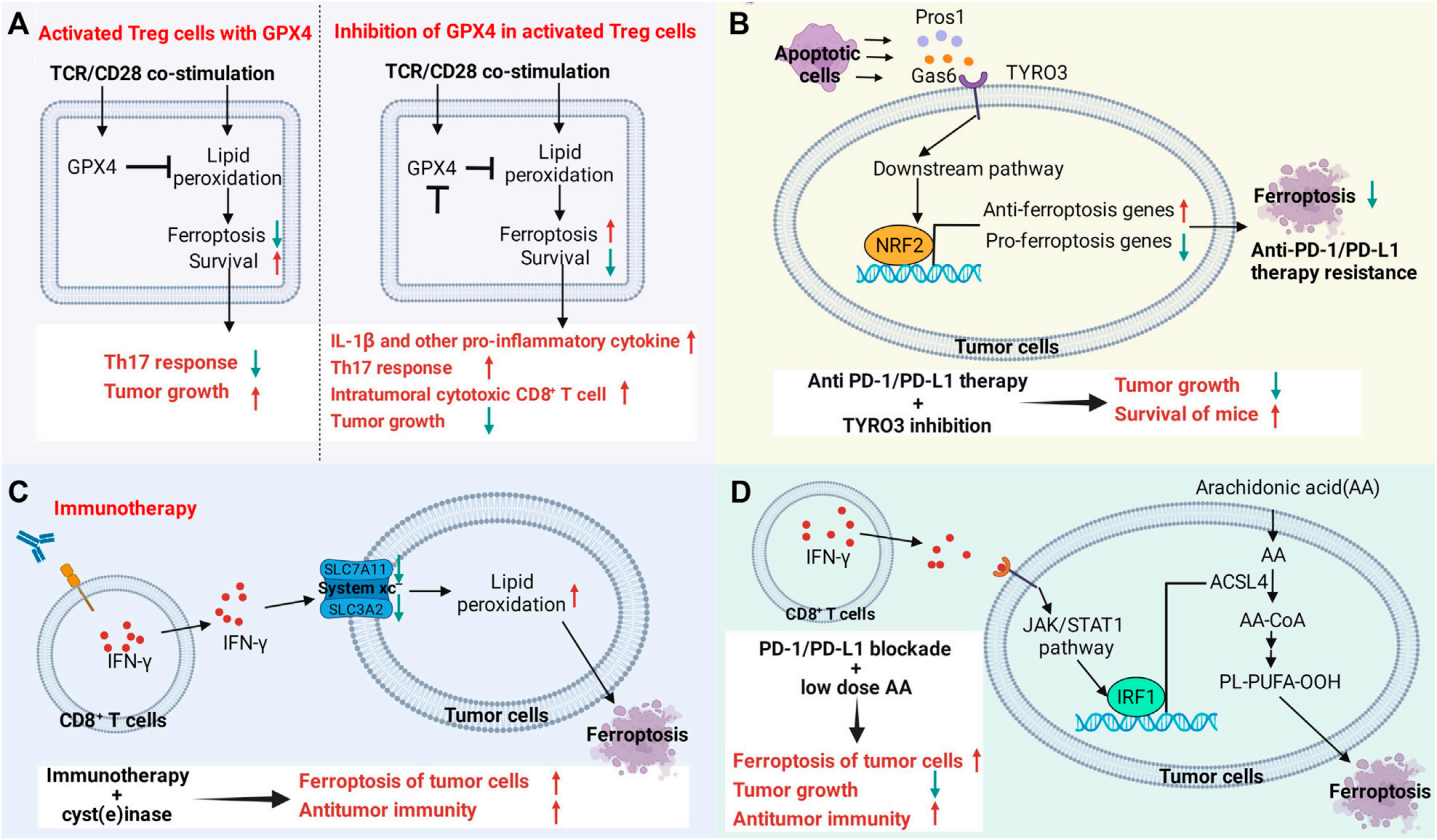
Targeting the ferroptosis pathway in immune cells or cancer cells reverses immunotherapy resistance or enhances therapeutic efficacy. **(A)** The ferroptosis signaling pathway in immune cells regulates antitumor immune function. Gpx4 protects activated Treg cells from lipid peroxidation and ferroptosis. Loss of Gpx4 leads to excessive accumulation of lipid peroxides and ferroptosis of Treg cells after TCR/CD28 co-stimulation. Gpx4-deficient Treg cells upregulate the production of IL-1β and TH17 responses, increasing the number and killing activity of intratumoral CD8^+^ T cells. Knockdown of Gpx4 in Treg cells inhibited tumor growth and simultaneously enhanced antitumor immunity. **(B)** TYRO3 expressed by tumor cells leads to resistance to anti-PD-1/PD-L1 therapy by inhibiting tumor ferroptosis. Some molecules produced by apoptotic cells in the tumor microenvironment activate the AKT/NRF2 axis after binding to TYRO3, thereby promoting the transcription of ferroptosis-inducing genes and inhibiting the expression of ferroptosis-inducing genes, leading to anti-PD-1/PD-L1 therapy resistance. Inhibition of TYRO3 promotes tumor ferroptosis and sensitizes resistant tumors to anti-PD-1 therapy. **(C,D)** CD8^+^ T cell-derived IFN-γ in the tumor microenvironment promotes lipid peroxidation and ferroptosis in tumor cells. Drugs that promote ferroptosis enhance the antitumor efficacy of immunotherapy. **(C)** IFN-γ promotes lipid peroxidation and ferroptosis in tumor cells by inhibiting the expression of SLC3A2 and SLC7A11. **(D)** IFN-γ activates the JAK/STAT1 signaling pathway in tumor cells, which in turn promotes the expression of ACSL4 through interferon regulatory factor 1 (IRF1). Supplementation with low-dose AA promotes ferroptosis in tumor cells and enhances the antitumor activity of checkpoint therapy.

IFN-γ synergistically promotes tumor ferroptosis along with fatty acids in the tumor microenvironment through ACSL4. It contributes to the upregulation of ACSL4 via the STAT1 and IRF1 signaling pathways, which also enhances the incorporation of AA into C16 and C18 acyl chain-containing phospholipids. Low-dose AA enhances anticancer immunity and the therapeutic efficacy of PD-L1 blockade by enhancing ferroptosis. The expression level of ACSL4 in bladder cancer and melanoma patients was positively correlated with their survival, which was also parallel to the expression levels of CD8A and IFN-γ and the T-cell signature. Cancer patients expressing a high level of ACSL4 who are treated with ICB therapy present higher overall survival and progression-free survival. Therefore, the ACSL4 signaling pathway that targets ferroptosis in the tumor microenvironment is favorable to enhance the therapeutic efficacy of ICB (Liao et al., 2022). The mechanism by which targeted ferroptosis in immunocytes or cancer cells reverses immunotherapeutic resistance or enhances treatment is shown in Figure 4.

## 5 Ferroptosis and Cancer Metastasis

Metastasis is a critical stage of tumor progression and remains a major challenge in treating cancer. Metastasis of various cancers, including prostate cancer (Norum and Nieder, 2017; Peng et al., 2017), triple-negative breast cancer (Gaffan et al., 2006; Pei et al., 2015), cervical cancer (Ferlay et al., 2013), causing a considerable number of patients to relapse after treatment. There is large heterogeneity between metastases and primary tumors. Due to the lack of effective prevention or treatment strategies (Li et al., 2016), the survival rate of patients after conversion is significantly reduced.A growing amount of evidence supports the involvement of ferroptosis in cancer metastasis. Here, we review the influence of ferroptosis as a regulator of cancer metastasis.

### 5.1 NF2

Neurofibromin 2 (NF2/Merlin) is a typical tumor suppressor encoded by NF2, which locates in the plasma membrane, cell cortex and cytoskeleton, linking extracellular signals with intracellular communication (Meng et al., 2021). Notably, mutations of NF2 are a major cause of neurofibromatosis type 2 and multiple malignancies, including mesothelioma, melanoma, breast cancer, and colorectal cancer (Ferlay et al., 2013; Petrilli and Fernández-Valle, 2016). The E-cadherin-Merlin-Hippo-YAP axis is frequently mutated in cancer, and malignant alterations of multiple members in this signaling axis all makes cancer cells sensitive to ferroptosis, and Merlin (NF2) is an important member of this axis (Wu et al., 2019). Once E-cadherin, Merlin, and Hippo are inhibited, the activity of YAP, a proto-oncogenic transcriptional coactivator, is enhanced, which further promotes ferroptosis by upregulating ferroptosis regulators such as ACSL4 and TFRC (Wu et al., 2019). E-cadherin-mediated cell-to-cell interactions activate the Hippo signaling pathway (Okada et al., 2005; Kim et al., 2011), in which NF2 and the kinase cascade consisting of MST1, MST2, LATS1, and LATS2 are involved. NF2 has been validated to activate the Hippo signaling pathway by inhibiting the CRL4^DCAF1^ complex (Li et al., 2012; Li et al., 2014).

The deficiency of NF2 often drives mesothelioma (Bueno et al., 2016). In xenograft models of athymic nude mice subcutaneously injected with shNT-GPX4-iKO cells and shNF2-GPX4-iKO cells, knockdown of NF2 upregulates TFRC, ACSL4 and nuclear YAP, and doxycycline-induced GPX4 knockdown is able to eliminate cancer lesions. NF2 is also involved in the regulation of metastasis. In the orthotopic mouse model of mesothelioma, shNF2-GPX4-iKO cells grew more aggressively than shNT-GPX4-iKO cells. Bioluminescence imaging showed multiple metastases in mice administered shNF2-GPX4-iKO cells, while no metastases were found in those administered shNT-GPX4-iKO cells, suggesting that NF2 deficiency stimulates cancer metastasis (Wu et al., 2019). Reduction of NF2 activity also promotes the efficient metastasis of breast cancer and melanoma cells (Pan, 2010; Lamar et al., 2012).

### 5.2 Regulators of EMT

Epithelial–mesenchymal transition (EMT) is a process in which epithelial cells lose their cell-cell adhesion capacity and acquire a morphology and intercellular phenotype similar to that of fibroblasts. EMT mediates tumor metastasis by enhancing the migratory and invasive abilities of tumor cells by generating cancer stem cells. In addition, EMT also leads to resistance to therapy. Regulation of these processes by EMT is stimulated by transcription factors such as ZEB1, SNAI1, and TWIST1. Therefore, they are potential targets for inhibiting metastasis and drug resistance (Chen et al., 2021a). Through a series of metastatic changes, including local invasion, intravasation, circulation, extravasation, formation of micro-metasatsis and overt colonization, cancer cells present different E/M phenotypes to struggle in the microenvironment (Yang J. et al., 2020). The latest evidence has shown that a high-mesenchymal cell state relies on the GPX signaling pathway. Therefore, inhibiting GPX4 leads to ferroptosis in cancer cells, thus suppressing metastasis. Metadherin (MTDH) is a newly discovered cancer-associated protein that promotes EMT, invasion and metastasis in various types of cancers, including breast cancer (El-Ashmawy et al., 2019; Jin et al., 2019). MTDH enhances cell sensitivity to ferroptosis by downregulating GPX4 and SLC3A2, reducing cysteine and GSH levels and enhancing glutamate levels. Interestingly, increasing the expression of SNAI1, TWIST1 or ZEB1 restored sensitivity to ferroptosis (Wu et al., 2019). ZEB1, a transcription factor associated with EMT-mediated tumor metastasis, has been shown to promote ferroptosis by directly inhibiting GPX4 activity, as well as in part through ZEB1-induced upregulation of PPARγ, a master regulator of hepatic lipid metabolism. ZEB1 has been shown to play an important role in cellular lipid metabolism, which regulates lipid uptake, accumulation, and mobilization, and affects EMT-related plasma membrane remodeling, which occurs from lipoxygenase-mediated PUFA oxidation place (Viswanathan et al., 2017). As a result, multiple regulators of EMT may be favorable to cell sensitivity to ferroptosis (Bi et al., 2019). The first step in EMT involves breaking the contacts between epithelial cells. ECAD-mediated intercellular interactions in epithelial cells prevent ferroptosis by modulating the intracellular Merlin-Hippo signaling pathway. EMT can antagonize the aforementioned signaling axis and then release the activity of the proto-oncogene transcriptional coactivator YAP to stimulate ferroptosis (Wu et al., 2019). These are possible mechanisms for EMT leading to high susceptibility to ferroptosis (Viswanathan et al., 2017).

### 5.3 xCT

Clinical studies have found that the recurrence rate of xCT-positive tumors is significantly higher than that of xCT-negative tumors, and the expression level of xCT is correlated with metastasis (Sugano et al., 2015). Overexpression of xCT has been identified as an indicator of poor prognosis in several types of cancers, including hepatocarcinoma (Chen et al., 2009; Lee et al., 2018). Malignant glioma cells kill surrounding neurons by xCT-released glutamate, thus providing a favorable condition for metastasis. The xCT antagonist S-(4)-CPG or sulfasalazine dose-dependently inhibits cancer cell migration by controlling the release of glutamate. Chronic inhibition of xCT-mediated glutamate release can effectively reduce tumor volume and the aggressiveness of tumor cells (Lyons et al., 2007). Therefore, the role of xCT in tumor invasion and metastasis should be considered.

### 5.4 HIF

The proliferation rate of cancer cells is much faster than that of the development of the vasculature system, leading to the lower oxygen supply rate in cancers than that of the oxygen consumption rate and thus causing hypoxia (Brahimi-Horn et al., 2007). The hypoxic microenvironment in cancer cells triggers the activation of hypoxia-related genes, including hypoxia-inducible factor (HIF) (Semenza, 2012). It is a major regulator of hypoxia that can enhance the invasion and metastasis of cancer cells (Rankin and Giaccia, 2016). HIF consists of an oxygen-labile α subunit (including HIF-1α, HIF-2α, and HIF-3α) and a constitutively expressed β subunit (HIF1β, also known as ARNT) (Keith et al., 2011). Under hypoxic conditions, HIF-1α promotes exosome discharge in gastric cancer cells and tissues. In addition, the positive feedback of HIF-1α/miR-301a-3p/PHD3 contributes to promoting the proliferation, invasion, migration, and EMT of gastric cancer cells (Xia et al., 2020). HIF-1α is upregulated in many types of cancers and is closely correlated with poor prognosis (Keith et al., 2011).

**FIGURE 5:**
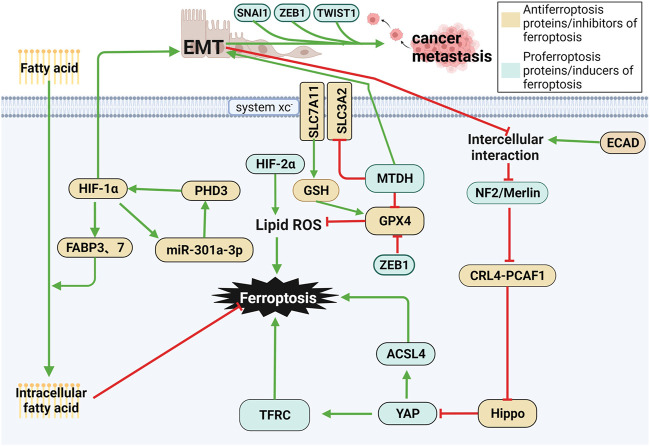
Ferroptosis and cancer metastasis. **(1)** Various changes in the E-cadherin-Merlin-Hippo-YAP axis are associated with ferroptosis. When E-cadherin, Merlin, and Hippo are inhibited, YAP is activated to further induce ferroptosis, while NF2/Merlin Deficiency drives cancer metastasis. **(2)** EMT is favorable to the survival of cancer cells and metastasis, which blocks E-cadherin-induced cell–cell interactions and activates YAP, thus leading to ferroptosis. MTDH contributes to ferroptosis by reducing intracellular GSH levels by downregulating GPX4 and SLC3A2. **(3)** HIF has a dual role in regulating ferroptosis in cancer cells. Activated HIF-2α upregulates lipid and iron-regulated genes and enhances lipid peroxidation of PUFAs, thus enhancing their sensitivity to ferroptosis. In contrast, it prevents ferroptosis in cancer cells by improving the cellular uptake of fatty acids and lipid storage by upregulating FABP3 and FABP7.

HIF has a dual role in regulating ferroptosis in cancer cells. Activated HIF-2α upregulates lipid- and iron-regulated genes in mouse CRC cells, thus enhancing their sensitivity to ferroptosis. In addition, activation of HIF-2α leads to ferroptosis by enhancing lipid peroxidation of PUFAs through irreversible cysteine oxidation (Singhal et al., 2021). However, in the fibrosarcoma cell line HT-1080, hypoxia-induced HIF-1α improves cellular uptake of fatty acids and lipid storage and inhibits subsequent lipid peroxidation and ferroptosis by upregulating fatty acid-binding proteins 3 (FABP3) and FABP7 (Yang et al., 2019). Therefore, HIF is of great significance in the regulation of ferroptosis, serving as a potential target for preventing cancer metastasis. The links between ferroptosis and tumor metastasis are shown in Figure 5.

### 5.5 Noncoding RNAs Regulate Ferroptosis During Metastasis

Previous studies have mainly focused on relevant genes, enzymes and signaling pathways associated with ferroptosis, including p53, GPX4, ACSL 4, SCL7A11, NFS1, etc. Recent studies have shown that some noncoding RNAs (e.g., miR-9 and miR-137) are also involved in the regulation of ferroptosis in cancer cells (Liu Y. et al., 2021), serving as oncogenes or tumor suppressor genes by indirectly mediating signaling pathways in the tumor micro-environment (Zhang et al., 2019). Here, we summarize the regulatory effect of RNAs on ferroptosis during carcinogenesis and cancer metastasis. Noncoding RNA modulate iron death in tumor metastasis is summarized in Table 3.

**TABLE 3:** Noncoding RNAs regulate ferroptosis in cancer metastasis.

RNAs	Targets	Functions in cancer metastasis	Referencs
Lnc-RNAs			
PVT1	miR-214	PVT1 promotes ferroptosis by downregulating miR-214, which promotes the metastasis of NSCLC, gastric cancer and oral squamous cell carcinoma	Lu et al. (2020)
ZFAS1	miR-150-5p	ZFAS1 inhibits ferroptosis by downregulating SLC38A1 by suppressing miR-150-5p level. MiR-150 is closely correlated with the metastasis of nasopharyngeal carcinoma	Liu et al. (2018), Yanni Yang et al. (2020)
MIR503HG	EMT-related proteins	Overexpression of MIR503HG inhibits cancer metastasis by downregulating EMT-related proteins like ZEB1 and N-cadherin	Qiu et al. (2019)
GAS5	miR-23a-3p	GAS5 upregulates PTEN by sponging miR-23a-3p, thus inhibiting osteosarcoma cell invasion via the PI3K/AKT pathway	Liu et al. (2020a)
Circ-RNAs			
TTBK2	miR-761 and miR-1283	CircTTBK2 promotes ferroptosis by modulating ITGB8 by sponging miR-761 in glioma. Knockdown of circ-TTBK2 inhibits proliferation, migration, invasion of glioma cells by mediating miR-1283 and CHD1	Han et al. (2020), H-Y Zhang et al. (2020)
RNAs	Targets	Functions in cancer metastasis	Refs
IL4R	miR-541-3p/GPX4 and miR-761	CircIL4R promotes ferroptosis in CRC cells via the miR-541-3p/GPX4 axis. It promotes the proliferation and metastasis of CRC by activating the PI3K/AKT signaling pathway via the miR-761/TRIM29/PHLPP1 axis	Xu et al. (2020), Tao Jiang et al. (2021)
mi-RNAs			
miR-103a-3p	GLS2 and TPD52	Knockdown of miR-103a-3p triggers ferroptosis in gastric cancer by downregulating GLS2. MiR-103a-3p promotes the metastasis of salivary adenoid cystic carcinoma by targeting TPD52	Niu et al. (2019), Fu et al. (2020)
miR-214-3p	ATF4	MiR-214 reduces the volume and weight of xenograft tumor tissues by enhancing Erastin-induced ferroptosis and downregulating ATF4. Plasma miR-214-3p level is significantly associated with tumor stage, recurrence and metastasis of nasopharyngeal carcinoma	Bai et al. (2020), Wang et al. (2020)
miR-137	SLC1A5 and KDM1A	MiR-137 negatively regulates ferroptosis by directly targeting glutamine transporter SLC1A5 in melanoma cells. Knockdown of miR-137-3p promotes the invasiveness of CRC by upregulating KDM1A/LSD1	Luo et al. (2018), Ding et al. (2021)
miR-23a-3p	DMT1	HUCB-MSCs-exosomes inhibits ferroptosis by downregulating DMT1 via miR-23a-3p. Knockdown of miR-23a promotes the metastasis of cutaneous melanoma	Guo et al. (2017), Song et al. (2021)

#### 5.5.1 Lnc-RNAs Regulate Ferroptosis During Metastasis

Preclinical studies have demonstrated the role of lnc-RNAs in the metastasis of various types of cancers by regulating ferroptosis via multiple mechanisms. For example, lncRNA PVT1 promotes ferroptosis by upregulating TFR1 and TP53 after targeting miR-214, forming a positive feedback loop of lncRNA PVT1/miR-214/p53 (Lu et al., 2020). It also triggers the metastasis of NSCLC (Qi and Li, 2020), gastric cancer (Niu et al., 2020) and oral squamous cell carcinoma (Zhu et al., 2020). The expression level of lncRNA ZFAS1 is positively correlated with that of SLC38A1, which is an important regulator of lipid peroxidation. Knockdown of lncRNA ZFAS1 inhibits ferroptosis by preventing intracellular lipid peroxidation by downregulating SLC38A1 (Yang Y. et al., 2020). MiR-150 is the target of lncRNA ZFAS1, which is an independent prognostic factor for nasopharyngeal carcinoma and is closely linked with its metastasis and poor prognosis (Liu et al., 2018).

#### 5.5.2 Circ-RNAs Regulate Ferroptosis During Metastasis

Circ-RNAs are also involved in regulation of ferroptosis during metastasis. CircTTBK2 promotes the metastasis of NSCLC by negatively regulating miR-761, which further inhibits ferroptosis by targeting ITGB8. Knockdown of circTTBK2 significantly alleviates the proliferation and invasion of glioma cells and induces ferroptosis (Zhang H. Y. et al., 2020). Compared with those of healthy volunteers, miR-761 levels in serum and tissues of NSCLC patients are both upregulated. The ectopic expression of miR-761 stimulates the proliferation and metastasis of H460 cells, and its knockdown reduces the proliferative and metastatic rates in H23 cells. The promotive effect of miR-761 on ferroptosis has been validated by relying on the targets ING4 and TIMP2 (Yan et al., 2015).

#### 5.5.3 miRNAs Regulate Ferroptosis During Metastasis

A growing number of miRNAs have been identified to be involved in ferroptosis. Dysregulated miR-214 is closely linked with osteolytic bone metastasis in breast cancer (Liu et al., 2017). Overexpression of premiR-214 stimulates erastin-induced ferroptosis by enhancing ROS levels and reducing GSH levels *in vitro*. SLC7A11/xCT is the target of miR-5096. The restoration of xCT inhibits miR-5096–induced ferroptosis and anticancer effects on human breast carcinoma cells by mediating lipid ROS, iron accumulation and GSH levels. A preclinical trial reported that miR-5096 contributes to inhibiting the colony formation, invasion and migration of cancer cells, while anti-miR-5096 significantly stimulates these carcinogenic features (Yadav et al., 2021). At present, the interaction among miRNAs, ferroptosis and cancer metastasis has not been fully elucidated, which requires further in-depth research.

## 6 Conclusion

The role of ferroptosis in cancer and the strategies of exploiting ferroptosis to overcome cancer drug resistance and treat metastasis have attracted the interest of many researchers over the past few years. As described in this study, the discovery and exploration of ferroptosis has opened up a new platform for the field of cancer therapy, and its clinical significance in cancer therapy resistance and metastasis is gradually emerging. The combined use of ferroptosis inducers can improve the efficacy of many FDA-approved anticancer drugs including platinum drugs, docetaxel, paclitaxel, temozolomide, sorafenib, and cetuximab, showing great potential for suppressing drug resistance. Furthermore, the induction of ferroptosis is also associated with the control of cancer metastasis. These findings raise high expectations for the role of ferroptosis in cancer treatment. However, there are more issues that need further clarification. Which chemotherapeutic agents can improve drug resistance by combining ferroptosis inducers? Is there anything in common between these drugs? How can potential adverse events due to ferroptosis be avoided? How to exploit the potential relationship between ferroptosis and cancer metastasis to prevent cancer metastasis? In order to further enhance the efficacy of anticancer drugs, overcome drug resistance, and inhibit cancer metastasis, more detailed studies on the mechanism of ferroptosis and the mechanism of ferroptosis-inducing agents combined with anticancer drugs are required.

## Glossary

DECR1: 2,4-Dienoyl-CoA reductase
5-FU: 5-Fluorouracil
5-hpete: 5-Hydroperoxyeicosatetraenoic acid
5-HETE: 5-Hydroxy-eicosatetraenoic acid
ALOX5: Arachidonate 5-lipoxygenase
ACAC: Acetyl-CoA carboxylase
ACSF2: Acyl-CoA synthetase family member 2
ACSL4: Acyl-CoA synthetase long-chain 4
AdA: Adrenic acid
AMPK: AMP-activated protein kinase
AR: Androgen receptor
ARE: antioxidant responsive element
ARIs: AR inhibitors
AA: Arachidonic acid
MRP5, ABCC5: ATP-dependent multidrug resistance protein-5
ATG: Autophagy-related
BFA: Brefeldin A
CBR1: Carbonyl reductase 1
CRPC: Castration-resistant prostate cancer
CTSB: Cathepsin B
CTCs: Circulating tumor cells
DDP: Cisplatin
CRR: Citrate clinically relevant radioresistant
CS: citrate synthase
(CRISPR)-Cas9: Clustered regularly interspaced short palindromic repeats
CoQ10: Coenzyme Q10
CRC: Colorectal cancer
NFS1: Cysteine desulfurase
cPLA2: Cytosolic phospholipase A2
DCs: Dendritic cells
DHA: Dihydroartemisinin
DPP4: Dipeptidyl-peptidase-4
DFS: Disease-free survival
EGFR-TKIs: EGFR-tyrosine kinase inhibitors
ETC: Electron carriers
EGFR: Epidermal growth factor receptor
EGFR-TKIs: Epidermal growth factor receptor-tyrosine kinase inhibitors
EMT: Epithelial–mesenchymal transition
FAO: Fatty acid β-oxidation
FABP3: Fatty acid-binding proteins 3
FTH1: Ferritin heavy polypeptide 1
FRGs: Ferroptosis-related genes
GSH: Glutathione
GPXs: Glutathione peroxidases
GCA: Golgicide A
HNC: Head and neck cancer
HCC: Hepatocellular carcinoma
hnRNPA1: Heterogeneous nuclear ribonucleoprotein A1
HMGB1: High mobility group protein 1
HDAC: Histone deacetylase
HIF: Hypoxia-inducible factor
ICB: Immune checkpoint blockade
IRF1: Interferon regulatory factor 1
IR: Ionizing radiation
ISCs: Iron-sulfur clusters
LA: Linoleic acid
LUAD: Lung adenocarcinoma
LPCAT3: Lysophosphatidylcholine Acyltransferase 3
MTDH: Metadherin
MT1G: Metallothionein-1G
MTs: Metallothioneins
MitoQ: Mitoquinone
MT: Mito-TEMPO
MUFAs: Monounsaturated fatty acids
NF2: Neurofibromatosis type 2
NADPH: Nicotinamide adenine dinucleotide phosphate
NSCLC: Non-small-cell lung cancer
OS: Overall survival
NOXs: NADPH oxidases
PC: Prostate cancer
PDAC: pancreatic ductal adenocarcinoma
PUFAs: polyunsaturated fatty acids
PRDXs: Peroxiredoxins
PLOH: Phospholipid alcohol
PLOOHs: Phospholipid hydroperoxides
PLs: Phospholipids
PSTK: Phosphoseryl-tRNA kinase.
PCA: Pancreatic cancer
RSL3: Ras-selective lethal 3
ROS: Reactive oxygen species
STAT3: Signal transducer and activator of transcription 3
SIRT6: Sirtuin 6
xCT: SLC7A11
SREBP: Sterol regulatory element-binding protein
STING1: stimulator of interferon response cGAMP interactor 1
SAS: Sulfasalazine
BH4: Tetrahydrobiopterin
TF: Transferrin
TFRC: Transferrin receptor
TGF-β1: Transforming growth factor-beta1
TCA: Tricarboxylic acid
TNBC: Triple-negative breast cancer
Tyro3: Tyrosine-protein kinase receptor
USP7: Ubiquitin-specific protease 7
